# Gene-expression molecular subtyping of triple-negative breast cancer tumours: importance of immune response

**DOI:** 10.1186/s13058-015-0550-y

**Published:** 2015-03-20

**Authors:** Pascal Jézéquel, Delphine Loussouarn, Catherine Guérin-Charbonnel, Loïc Campion, Antoine Vanier, Wilfried Gouraud, Hamza Lasla, Catherine Guette, Isabelle Valo, Véronique Verrièle, Mario Campone

**Affiliations:** 1 Unité Mixte de Génomique du Cancer, Institut de Cancérologie de l’Ouest - site René Gauducheau, Bd J. Monod, 44805 Saint Herblain Cedex, France; 2 Unité de Bioinfomique, Institut de Cancérologie de l’Ouest - site René Gauducheau, Bd J. Monod, 44805 Saint Herblain Cedex, France; 3 Département de Biopathologie, Institut de Cancérologie de l’Ouest - site René Gauducheau, Bd J. Monod, 44805 Saint Herblain Cedex, France; 4 INSERM U892, IRT-UN, 8 quai Moncousu, 44007 Nantes Cedex, France; 5 Service d’Anatomie Pathologique B, Hôpital G&R Laënnec, Bd J. Monod, 44805 Saint Herblain, France; 6 Unité de Biostatistique, Institut de Cancérologie de l’Ouest - site René Gauducheau, Bd J. Monod, 44805 Saint Herblain Cedex, France; 7 Sorbonne Universités, UPMC University, Département de Biostatistique, 4 place Jussieu, 75005 Paris, France; 8 AP-HP, University Hospitals Pitié-Salpêtrière Charles-Foix, Département de Biostatistique, Santé Publique et Informatique Médicale, 47-83 Bd de l’Hôpital, 75013 Paris, France; 9 Institut de Cancérologie de l’Ouest - site Paul Papin, 2 rue Moll, 49933 Angers Cedex 9, France; 10 Laboratoire d’Anatomie Pathologique, Institut de Cancérologie de l’Ouest - site Paul Papin, 2 rue Moll, 49933 Angers Cedex 9, France; 11 Service d’Oncologie Médicale, Institut de Cancérologie de l’Ouest - site René Gauducheau, Bd J. Monod, 44805 Saint Herblain Cedex, France

## Abstract

**Introduction:**

Triple-negative breast cancers need to be refined in order to identify therapeutic subgroups of patients.

**Methods:**

We conducted an unsupervised analysis of microarray gene-expression profiles of 107 triple-negative breast cancer patients and undertook robust functional annotation of the molecular entities found by means of numerous approaches including immunohistochemistry and gene-expression signatures. A triple-negative external cohort (*n* = 87) was used for validation.

**Results:**

Fuzzy clustering separated triple-negative tumours into three clusters: C1 (22.4%), C2 (44.9%) and C3 (32.7%). C1 patients were older (mean = 64.6 years) than C2 (mean = 56.8 years; *P* = 0.03) and C3 patients (mean = 51.9 years; *P* = 0.0004). Histological grade and Nottingham prognostic index were higher in C2 and C3 than in C1 (*P* < 0.0001 for both comparisons). Significant event-free survival (*P* = 0.03) was found according to cluster membership: patients belonging to C3 had a better outcome than patients in C1 (*P* = 0.01) and C2 (*P* = 0.02). Event-free survival analysis results were confirmed when our cohort was pooled with the external cohort (*n* = 194; *P* = 0.01). Functional annotation showed that 22% of triple-negative patients were not basal-like (C1). C1 was enriched in luminal subtypes and positive androgen receptor (luminal androgen receptor). C2 could be considered as an almost pure basal-like cluster. C3, enriched in basal-like subtypes but to a lesser extent, included 26% of claudin-low subtypes. Dissection of immune response showed that high immune response and low M2-like macrophages were a hallmark of C3, and that these patients had a better event-free survival than C2 patients, characterized by low immune response and high M2-like macrophages: *P* = 0.02 for our cohort, and *P* = 0.03 for pooled cohorts.

**Conclusions:**

We identified three subtypes of triple-negative patients: luminal androgen receptor (22%), basal-like with low immune response and high M2-like macrophages (45%), and basal-enriched with high immune response and low M2-like macrophages (33%). We noted out that macrophages and other immune effectors offer a variety of therapeutic targets in breast cancer, and particularly in triple-negative basal-like tumours. Furthermore, we showed that CK5 antibody was better suited than CK5/6 antibody to subtype triple-negative patients.

**Electronic supplementary material:**

The online version of this article (doi:10.1186/s13058-015-0550-y) contains supplementary material, which is available to authorized users.

## Introduction

Breast cancer heterogeneity makes it difficult to bring personalized medicine into the clinic. For many years, research has been aimed at deciphering molecular presentation of this disease to identify subgroups of patients with clinical significance, such as prognosis or response to therapy, in order to optimize patient management. Immunohistochemistry (IHC)-typed triple-negative (TN) tumours, which represent 12 to 17% of primary breast cancer, are among the most aggressive and deadly breast cancer subtypes [1]. The lack of oestrogen, progesterone and HER2 receptors makes its therapeutic management optimisation difficult. Furthermore, TN breast cancer tumours are also known to be heterogeneous. The era of large-scale science, which is linked both to recent technological advances and to the availability of full genetic information, has boosted the research for new biomarkers and molecular subtyping. In 2000 and 2001, breast tumour gene-expression profiling (GEP) revealed five intrinsic subtypes: luminal A, luminal B, HER2-enriched (HER2-E), basal-like and normal breast-like [2,3]. Luminal A tumours were associated with a better prognosis in comparison with other subtypes, which displayed similar bad prognoses. Luminal and HER2-E subtypes defined subgroups of patients who could receive targeted therapies (hormonotherapy and trastuzumab, respectively). Basal-like tumours are often mixed up with TN tumours; the main difference comes from the typing method (GEP for basal-like and IHC for TN). Comparative studies showed that not all TN tumours are identified as basal-like (approximately 80%), and not all basal-like tumours are TN [1,4]. Both types affect younger patients, and are linked to a bad prognosis and no possibility of targeted therapy. Other works refined TN subtyping and identified a new molecular entity, named claudin-low, characterized by low expression of cell-cell adhesion cluster containing claudin 3, 4, 7 and E-cadherin, luminal and proliferation-associated genes, enrichment in epithelial-to-mesenchymal transition (EMT) features, immune system responses, and stem cell-associated biological processes [5,6]. No prognostic difference was noted between basal-like and claudin-low patients [6]. An interferon-rich subgroup, characterized by high expression of interferon-regulated genes, was further discovered [7]. Gene Ontology (GO) categories of these genes underlined “immune response” and “defense response”. Later, six molecular subtypes were also identified in TN: basal-like 1, basal-like 2, immunomodulatory, mesenchymal-like, mesenchymal stem-like, and luminal androgen receptor (LAR) [8]. Today, heterogeneity within basal-like and TN is still controversial and requires further research, including new cohorts, to understand the complexity of the disease, and to identify molecular drivers that could be therapeutically targeted [1,6].

In this study, we conducted an unsupervised analysis of a new cohort of 107 TN breast cancer patients and undertook robust functional annotation of the main molecular entities found by means of numerous complementary approaches.

## Methods

### Patients

The bi-centric studied cohort retrospectively included 107 randomly selected women whose primary breast tumours lacked immunohistochemical expression of oestrogen receptor (ER), progesterone receptor (PR) and HER2; hence subtyped as TN tumours. Patients were diagnosed and treated primarily between 1998 and 2007 at the Institut de Cancérologie de l'Ouest – René Gauducheau (*n* = 65) and the Institut de Cancérologie de l'Ouest – Paul Papin (*n* = 42). Of the 107 patients, 44 relapsed and 63 were disease-free after the follow-up period (median follow-up = 7 years). All patients showed no evidence of relapse at the time of diagnosis. None had received chemotherapy, endocrine therapy or radiation therapy prior to surgery. Treatment decisions and follow-up processes were based solely on international recommendations. The follow-up data of patients included clinical examination and mammography every 6 months for 2 years and annually thereafter. Informed consent was obtained from patients to use their surgical specimens and clinicopathological data for research purposes, as required by the French Committee for the Protection of Human Subjects (CCPPRB). Ouest IV – Nantes CCPPRB approved use of tumour tissues for this study (6 May 2013: n°. 357/2013). Collection of tumours was approved by French Minister of higher education and research (n°. AC-2008-141). This study did not need additional ethical approval.

### Tumour tissues

All tumour tissue samples were surgically collected and processed in two parts by a pathologist. The first part was fixed in 10% neutral buffered formalin for standard histological analysis and IHC. The second part was immediately macrodissected, snap-frozen in liquid nitrogen and stored until RNA extraction.

### RNA extraction

Total RNA was prepared following standard protocols then treated with Dnase I using the RNeasy column purification system (Qiagen, Courtaboeuf, France). Assessment of RNA quality, integrity and purity was done through a Bionalyser 2100 (Agilent Technologies, Palo Alto, CA, USA). RNA samples were considered for further analysis only if they had distinct 28 S and 18 S ribosomal peaks.

### Gene expression profiling

Gene expression analysis was performed using Affymetrix® Human Genome U133 Plus 2.0 Arrays (Affymetrix®, Santa Clara, CA, USA) measuring over 43,000 transcripts representing over 20,000 human genes. cRNA synthesis and labelling, as well as chip hybridisation, washing and image scanning were performed according to the manufacturer’s protocol. All microarrays complied with quality criteria. Microarray and patient clinical data have been deposited in the Gene Expression Omnibus (GEO) under the GSE58812 accession number.

### Bioinformatics

#### Internal and external data pre-processing

Bioclinical and expression data of the external cohort used for validation were available from the GEO, accession number GSE21653 [9,10]. For both our cohort and the external cohort, the Affymetrix® CEL files (raw data) were MAS5-normalized in the Affymetrix Expression Console (v1.3.1) and then log2-transformed.

### Unsupervised analysis

To organise data into groups with the same underlying molecular characteristics, we performed clustering analysis based on the 5% most variable probe sets (*n* = 2,734) by means of the fuzzy clustering method. Fuzzy clustering permits each patient to have a probability of membership to each cluster along with the cluster number, with the sum of all cluster membership probabilities being 1 for any patient. Briefly, the method is based on the minimization of ${\displaystyle {\sum}_{k=1}^n\frac{{\displaystyle {\sum}_{i,j\kern0.5em }}u{\left(i,k\right)}^ru{\left(j,k\right)}^rd\left(i,j\right)}{2{\displaystyle {\sum}_j}\kern0.5em u{\left(j,k\right)}^r}}$, where *n* is the number of clusters, i and j represent the patients, u(i,k) is the membership of patient i for cluster k, r is the fuzziness index and d(i,j) is the dissimilarity between patients i and j (centred Pearson distance in our study). To decide how many clusters were present in our data we calculated two indices, the Dunn index and the Calinski and Harabasz index, considering two to 10 clusters, but we also performed a hierarchical clustering to visualise the partition and study the concordance of both clustering methods. All computations showed either two or three clusters (similar results for both numbers). It was decided to create three clusters and study the membership probabilities.

### Cluster functional annotation

To annotate the clusters found, we used clinicopathologic characteristics, 10 IHC markers, 17 gene-expression signatures (GES), GO biological process terms enrichment, and intuitive single gene-expression approach. Differences among the three clusters for the different characteristics were assessed by analysis of variance (along with Tukey’s HSD (honest significant differences) test in case of significance) for continuous variables, Fisher’s exact test for categorical variables, and Cox regression model or Kaplan-Meier survival curves (along with logrank test) for survival data. Correlations between GES were measured by Pearson’s correlation coefficient.

### Tissue microarrays and imunohistochemistry

#### Tissue microarray construction

Hemalun-eosin-safran (HES)-stained sections were reviewed and the area of interest was marked out on the slide. For each case, three representative tumour areas were selected from the HES-stained slide of the paraffin-embedded donor block. Using a tissue arrayer (Beecher Instruments, Sun Prairie, WI, USA), 1-mm diameter cores of breast cancer tissue were punched out from the paraffin-embedded tissue block and placed into recipient paraffin blocks. A control tissue sample (normal breast) was included in each recipient paraffin block. Before immunostaining, HES-stained slides of the final array blocks were examined to confirm the representative areas of the tumours in comparison to the original HES-stained section.

### Immunohistochemistry and scoring

Sections of paraffin-embedded tissue microarray blocks (4 μm thick) were deparaffinized in xylene and rehydrated through a graded series of alcohol. IHC was performed using the EnVision Detection Systems (Dako, Les Ulis, France) according to the manufacturer’s instructions; 3,3′-diaminobenzidine was used as a chromogen. The sections were counterstained with haematoxylin. Details of the antigen retrieval technique and dilution of the primary antibodies (CK5/6, CK5, HER1, androgen receptor (AR) Ki-67, FOXA1, E-cadherin, claudin 3, claudin 4 and claudin 7) are described in Additional file 1. Immunostaining results were assessed by a pathologist in a blinded manner. Immunoreactivity of each target in the tissue microarray cores was scored as a percentage of cells stained and/or staining intensity. Immunohistochemical staining interpretation is displayed in Additional file 2.

### Gene-expression signatures

Seventeen GES were used to annotate the three clusters: three single sample predictors (SSPs) (Sorlie’s SSP, Hu’s SSP and Parker’s SSP (PAM50)), proliferation score, a subtyping tool for TN breast cancer (TNBCtype), Teschendorff’s GES, vascular endothelial growth factor (VEGF) profile, glycolysis profile, claudin-low signature and seven immune metagenes [6-8,11-16]. Furthermore, in order to discriminate macrophages into M1-like or M2-like subpopulations, which could not be identified by Rody’s HCK metagene, we established an M2/M1 GES based on Beyer’s microarray data (GSE35449) [17]. The significance analysis of microarrays (SAM) method was applied to find the probes differentially expressed between M1 and M2 populations, and genes for which all corresponding probes exhibited a *Q*-value of 0% were retained. The GES was then computed in our cohort as the weighted mean of the genes retained, weights being +1 or −1, depending on the expression of each gene in M2 relative to M1. When a gene was represented by multiple probe sets, the median of the different probe sets was taken as the unique value for the gene. Methods are summarized in Additional file 3.

### Single gene-expression intuitive approach

An intuitive approach was also used to annotate patient clusters. Based on breast cancer transcriptomic studies, we selected representative genes (*n* = 49) of molecular subtypes (luminal, HER2-E, basal-like, claudin-low) and biological processes (proliferation, breast stem cells, EMT, cell migration, immune system response and angiogenesis) [6,18]. Their expressions were compared in function of clusters.

### Gene ontology biological process terms enrichment

Functional annotation of each cluster through biological process analysis was performed using DAVID bioinformatics resources 6.7 (the Database for Annotation, Visualization, and Integrated Discovery), Gorilla and ToppGene web tools to discover the GO categories with significantly enriched gene numbers [19-22]. Two methods were used to select genes differentially expressed across the clusters. The SAM method was performed to obtain lists of genes with significantly different expression between clusters (one versus the others and two-by-two); genes for which all corresponding probe sets had a *Q*-value of 0% were retained. In addition to the SAM method, expression of the 2,734 most variable probe sets was represented on a heatmap, with patients ordered according to the fuzzy clusters. Ward hierarchical clustering was performed on the probe sets, and clusters of corresponding genes with visually differential expression among the clusters of patients were retained.

### Unsupervised external validation process

The same global process as the one applied to our cohort was also applied to the TN patients of the external cohort (GSE21653) to validate unsupervised analysis results. Moreover, the prediction analysis for microarrays (PAM) method was used to predict clusters obtained with our cohort - the predictor was trained on our cohort and then applied to GSE21653 TN patients; the predict clusters were then compared to the ones obtained by fuzzy clustering.

### Statistical analysis

All statistical analyses were performed with R [23] and the packages amap, cluster, ggplots, grid, pamr, samr, survival. All *P*-values are two-sided; *P*-values less than 5% are considered significant.

## Results

### External data selection and pre-processing

We selected the GSE21653 cohort for external validation because the status of ER, PR and HER2 were available and gene expressions were measured with the same DNAchip as for our cohort. Of the 266 patients that composed the GSE21653 cohort, 87 patients were TN.

### Unsupervised analysis

**Figure 1:**
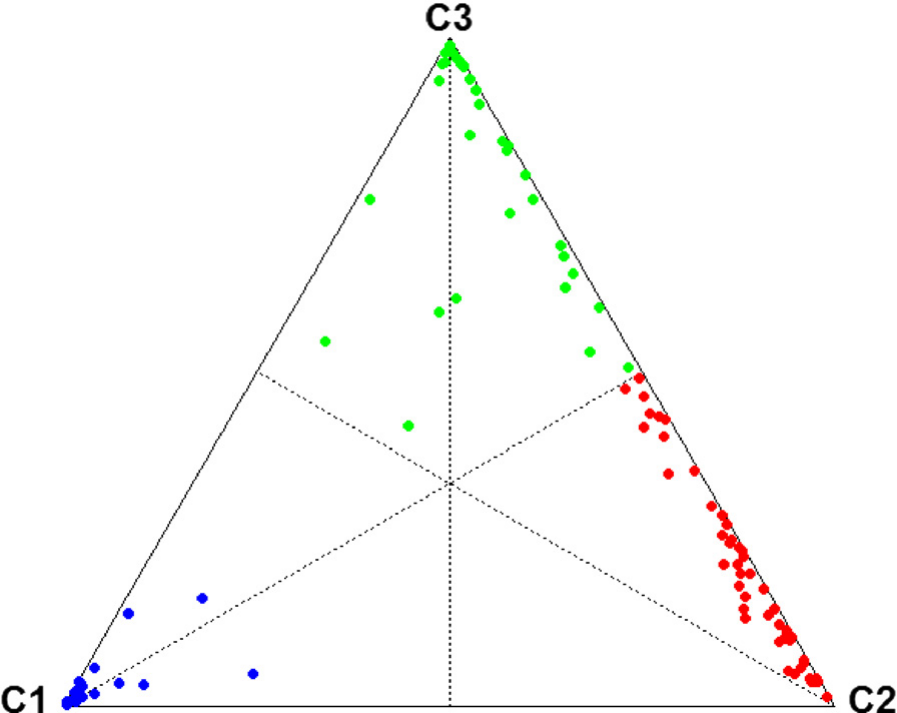
**Fuzzy clustering of 107 triple-negative breast cancer patients.** Distribution of patients based on probability of belonging to cluster C1 (blue), C2 (red) and C3 (green). Each vertex of the triangle represents a cluster and each point represents a patient, placed as the barycentre of the triangle, weights being the probabilities of belonging to each of the clusters. Hence, the closer a point is to one of the vertices, the greater is the probability of the patient belonging to the corresponding cluster.

We chose a fuzzy clustering method to investigate molecular differences among our TN cohort because it keeps the possibility for each patient to belong to multiple clusters at the same time but with different “degrees of membership”. Fuzzy clustering separated TN tumours into three clusters, named C1 (*n* = 24; 22.4%), C2 (*n* = 48; 44.9%) and C3 (*n* = 35; 32.7%) (Figure 1). Distribution of samples in all three clusters was independent of a patient’s origin (*n* = 2) (*P* = 0.25). Fuzzy clustering probabilities demonstrated that C1 was more robust than C2 and C3 (C1: minimum of probabilities = 73%, median = 98%, 20 C1 patients (83%) with probability >90%; C2: minimum of probabilities = 49%, median = 79%, 36 C2 patients (75%) with probability <90%; C3: minimum of probabilities = 42%, median = 85%, 19 C3 patients (54%) with probability <90%) and that C2 and C3 were very close. If we consider only the C2 and C3 patients, the smallest difference between the probability of belonging to C2 and the one of belonging to C3 was only 1%; for 11 patients (13%), this difference was below 20%.

### Cluster functional annotation

Cluster functional annotation results are detailed in the five following sections: clinicopathologic characteristics, IHC, GES, single gene-expression intuitive approach and GO biological process terms enrichment.

#### *Clinicopathologic characteristics*

**Figure 2:**
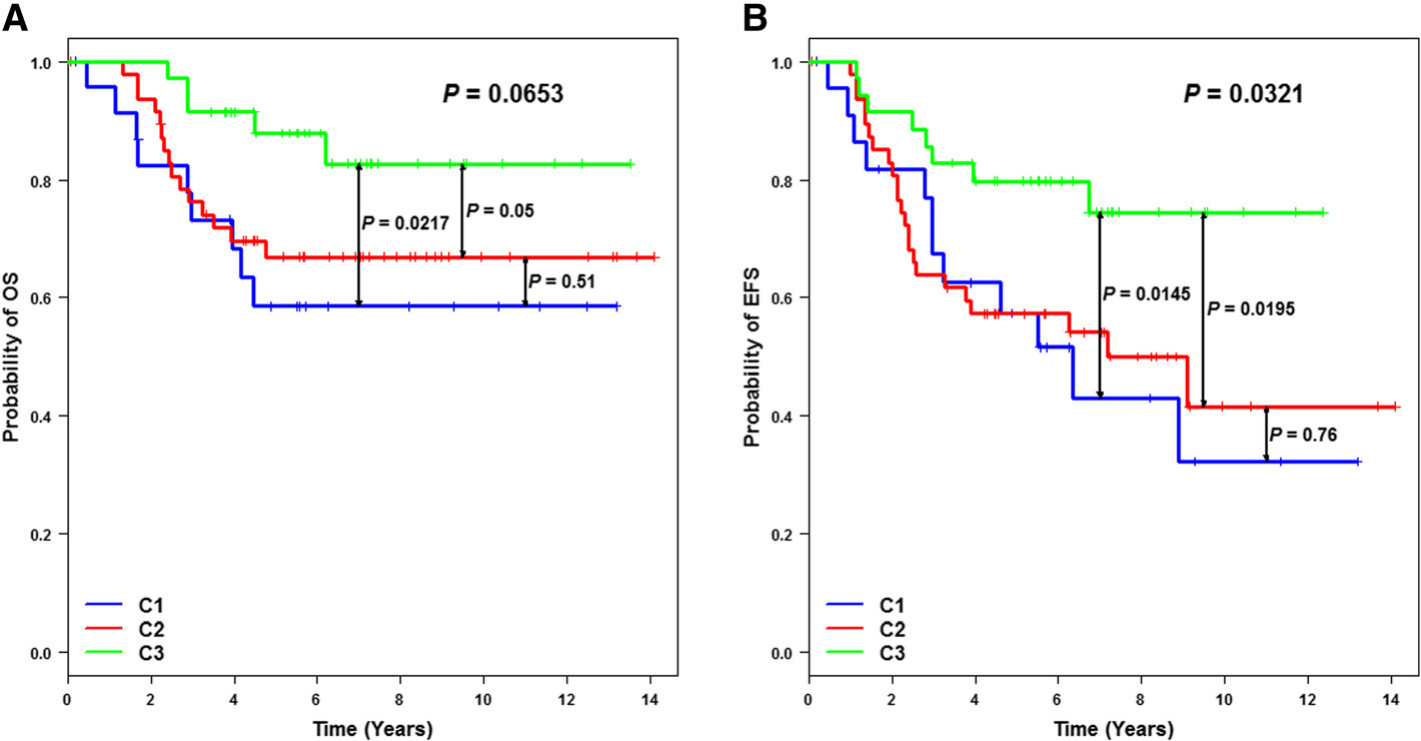
**Kaplan-Meier analyses of 107 triple-negative breast cancer patients based on fuzzy-clustering partition. (A)** Overall survival (OS) analysis shows that C3 patients have a better outcome than C1 (*P* = 0.0217) and C2 patients (*P* = 0.05). **(B)** Event-free survival (EFS) analysis shows the same result: C3/C1, *P* = 0.0145; C3/C2, *P* = 0.0195.

We have selected 107 breast carcinomas that were shown to lack IHC expression of ER, PR and HER2. The clinicopathologic characteristics of these 107 tumours are displayed in Table 1. Patients belonging to C1 were older (mean = 64.6 years) than patients included in C2 (mean = 56.8 years; *P* = 0.03) and in C3 (mean = 51.9 years; *P* = 0.0004). No significant age difference was observed between C2 and C3 (*P* = 0.17). Histological grade and Nottingham prognostic index (NPI) were higher in C2 and C3 than in C1. These results showed that C1 was different from C2 and C3, and that C1 seemed to be characterized by a less aggressive illness according to age, histological grade and NPI. A significant event-free survival (EFS) (*P* = 0.0321) and a trend for overall survival (OS) (*P* = 0.0653) were found according to cluster membership (Figure 2). Patients belonging to C3 had a better EFS than patients in C1 (*P* = 0.0145) and C2 (*P* = 0.0195). No outcome difference existed between C1 and C2 (*P* = 0.76). EFS results were confirmed when our cohort was pooled with the GSE21653 TN patients (*P* = 0.01) (Additional file 4). Patients belonging to C3 had a better EFS than patients in C1 (*P* = 0.03) and C2 (*P* = 0.002), and no outcome difference was observed between C1 and C2 (*P* = 0.45). One main reason might explain why patients belonging to C1 had a poor prognosis; C1 included numerous PAM50 luminal subtypes (10 out of 24; mainly luminal B (A, *n* = 1; B, *n* = 9)), which are characterized by a poor outcome. Furthermore, these luminal patients did not receive hormonotherapy. The same observation was made for GSE21653 and the equivalent cluster C1’, which included several luminal patients (7/27 (A, *n* = 1; B, *n* = 6)).

**Table 1:** **Clinicopathologic characteristics of the studies triple-negative tumours**

**Variable**	**All**	**Cluster 1**	**Cluster 2**	**Cluster 3**	***P*** **-value**
	**(** ***n*** **= 107)**	**(** ***n*** **= 24)**	**(** ***n*** **= 48)**	**(** ***n*** **= 35)**	
Age (years; mean ± SD)	56.9 ± 12.8	64.6 ± 10.6	56.8 ± 12.9	51.9 ± 11.7	0.0006
SBR grade					
1	2	2	0	0	
2	14	10	1	3	
3	91	12	47	32	<0.0001
Tumour size (mm; mean ± SD)	22.3 ± 12.7	27.3 ± 18.3	22.8 ± 12.1	18.5 ± 7.0	0.184
Nodal status					
0	77	17	38	22	
1	30	7	10	13	0.266
NPI					
1	13	9	1	3	
2	71	8	40	23	
3	22	6	7	9	<0.0001
Radiotherapy					
No	4	2	1	1	
Yes	103	22	47	34	0.436
Adjuvant therapy					
No	12	6	4	2	
Yes	95	18	44	33	0.071
Hormonotherapy					
No	106	23	48	35	
Yes	1	1	0	0	0.224

NPI, Nottingham prognostic index; SBR, Scarff Bloom Richardson.

#### Immunohistochemistry

Basal-like phenotype, as defined by Nielsen and colleagues [24] (ER negative and HER2 negative, and CK5/6 and/or HER1 positive) was found in 50% of C1 patients (the same result was found with CK5), and 71% or 92% in C2 + C3 patients by means of CK5/6 or CK5, respectively. Our results showed that CK5 IHC expression was more concordant with gene expression profiling results than CK5/6: 92% of C2 + C3 patients were typed as basal-like by means of CK5 and/or HER1 positivity and 71% by means of CK5/6 and/or HER1 positivity. Furthermore, considering CK5 and CK5/6 alone, 83% of C2 + C3 patients showed positivity for CK5 but only 34% had positivity for CK5/6 (*P* = 0.0019). Hence, CK5 should be used to demonstrate basal-like characteristics, as underlined by a few studies [25,26]. Briefly, functional annotation of fuzzy cluster results by means of IHC markers showed that C1 was not a basal-like cluster (AR and FOXA1 positive, low Ki-67 expression) and, on the contrary, that C2 and C3 were basal-like (CK5 and/or HER1 positive, high Ki-67 expression) (Table 2). C2 and C3 could not be distinguished based on IHC claudin-low markers (claudin 3, 4, 7 and E-cadherin). No statistical link was found between low claudin 3, 4 or 7 and claudin-low subtype as defined by Prat (*P* = 0.99, *P* = 0.56, *P* = 0.35, respectively) or between IHC claudin-low (claudin 3, 4, 7 negative and/or E-cadherin negative) and claudin-low (*P* = 0.52) [6].

**Table 2:** **Functional annotation of fuzzy clusters by means of immunohistochemistry**

**Marker interpretation**	**C1**	**C2**	**C3**	***P*** **-value**
	**(** ***n*** **= 24)**	**(** ***n*** **= 48)**	**(** ***n*** **= 35)**	
CK5/6 and/or HER1 positive	50%	71%		0.077
CK5 and/or HER1 positive	50%	92%		<10^−5^
Ki-67 positive	29%	87%		2.10^−7^
AR positive	73%	5%		<10^−9^
FOXA1 positive	73%	5%		7.10^−13^
E-cadherin positive	64%	48%		0.197
	-	42%	56%	0.236
Claudin 3 positive	38%	59%		0.085
	-	58%	60%	0.879
Claudin 4 positive	91%	93%		0.672
	-	92%	94%	0.990
Claudin 7 positive	18%	12%		0.485
	-	12%	11%	0.990
Claudin 3, 4, 7 negative and/or E-cadherin negative	38%	52%		0.261
	-	58%	43%	0.163

#### Gene-expression signatures

**Figure 3:**
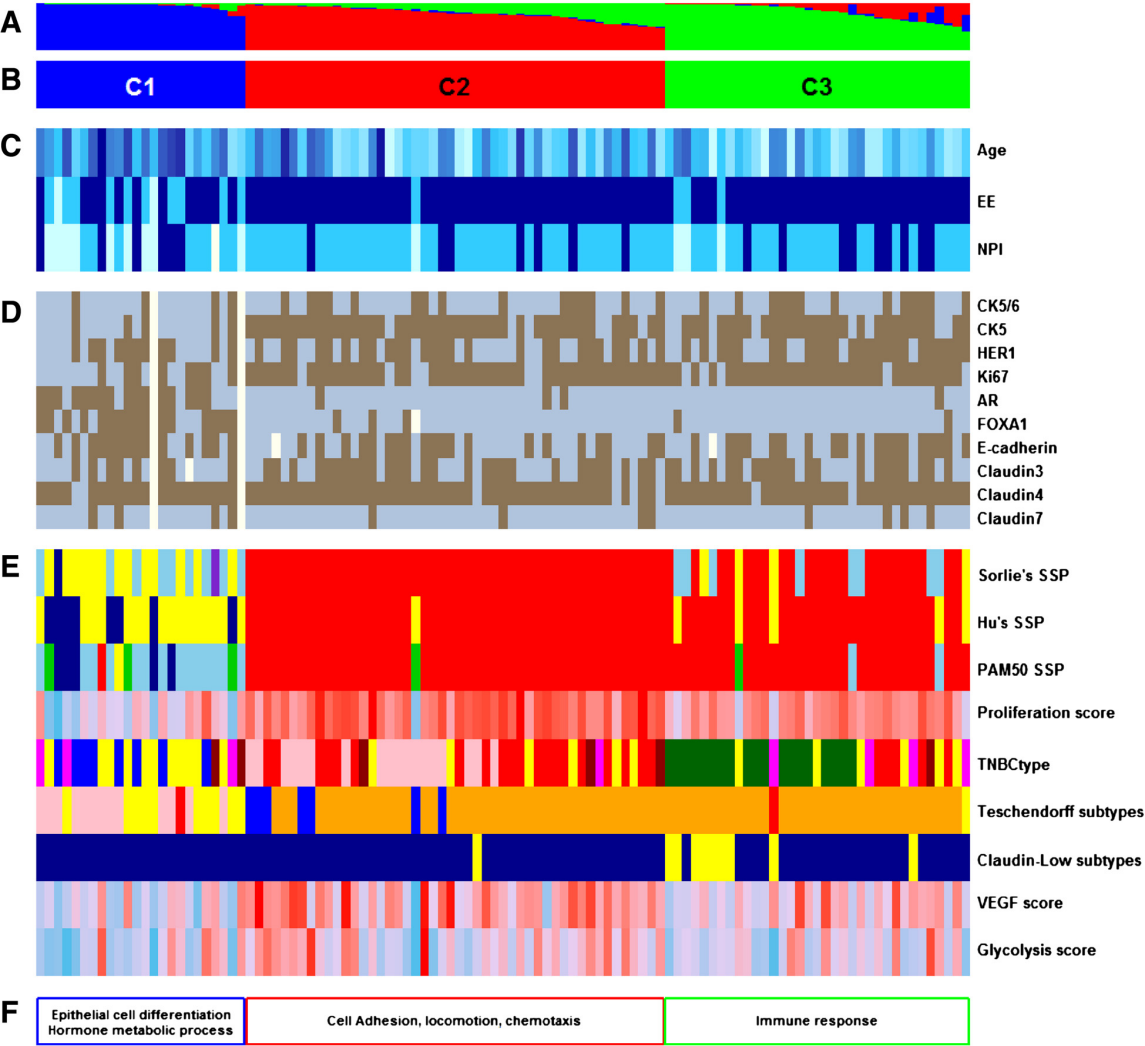
**Functional annotations of the clustering results of our cohort. (A)** Fuzzy-clustering probability of belonging to clusters, from 0 to 1. **(B)** Cluster numbering scheme. **(C)** Clinicopathologic characteristics with significant differences between clusters. Age as a continuous colour scale, from 28 years old (pale turquoise) to 85 years old (dark blue); Elston-Ellis (EE) histological grades 1 (pale turquoise), 2 (deep sky blue), and 3 (dark blue); Nottingham prognostic index (NPI) 1 (pale turquoise), 2 (deep sky blue), and 3 (darkblue). **(D)** Immunohistochemistry results for ten markers: positive (brown) and negative (blue). **(E)** Molecular subtyping by means of nine gene expression signatures (GES): three single sample predictors (SSPs) - luminal A (dark blue), luminal B (sky blue), HER2-E (purple), basal-like (red), normal breast-like (green) or unclassified (yellow); proliferation (continuous colour scale from minimum (6.59; deep sky blue) to maximum (11.05; red)); TNBCtype (basal-like 1 (red), basal-like 2 (dark red), immunomodulatory (dark green), mesenchymal-like (pink), mesenchymal stem-like (magenta), luminal androgen receptor (blue) and unclassified tumours (yellow)); Teschendorff’s GES (cell cycle (blue), cell cycle and immune response (orange), extracellular matrix (green), immune response (red), steroid hormone response (pink) and unclassified (yellow)); claudin-low (claudin-low (yellow), other (darkblue)); vascular endothelial growth factor (VEGF; continuous colour scale, from minimum (8.13; deep sky blue) to maximum (10.67; red)); and glycolysis (continuous colour scale, from minimum (9.63; deepskyblue) to maximum (11.88; red)). **(F)** Gene ontology biological process terms enrichment. Missing values are in white.

GES subtyping results are discussed below and detailed in Table 3 and Figure 3.

**Table 3:** **Gene-expression profiling of the three clusters**

**GES name**		**Cluster 1**	**Cluster 2**	**Cluster 3**	***P*** **-value**
		**(** ***n*** **= 24)**	**(** ***n*** **= 48)**	**(** ***n*** **= 35)**	
Sorlie’s SSP	Basal-like	0	48	23	<0.0001
	HER2-E	1	0	0	
	Luminal A	1	0	0	
	Luminal B	9	0	8	
	Unclassified	13	0	4	
Hu’s SSP	Basal-like	0	47	30	<0.0001
	HER2-E	0	0	0	
	Luminal A	8	0	0	
	Luminal B	0	0	0	
	Unclassified	16	1	5	
Parker’s SSP = PAM50	Basal-like	1	47	32	<0.0001
	HER2-E	0	0	0	
	Luminal A	5	0	0	
	Luminal B	14	0	2	
	NBL	3	1	1	
	Unclassified	1	0	0	
Proliferation score (mean ± sd)		8.64 ± 0.97	10.22 ± 0.63	9.74 ± 0.57	<0.0001
TNBCtype	BL1	0	20	5	<0.0001
	BL2	2	3	1	
	IM	0	0	19	
	LAR	8	0	0	
	M	0	18	0	
	MSL	3	1	4	
	Unclassified	11	6	6	
Teschendorff’s GES	CC+	0	7	0	
	CC+/IR+	0	41	33	
	IR+	1	0	1	
	SR+	13	0	0	
	Unclassified	10	0	1	
VEGF profile (mean ± SD)		9.20 ± 0.49	9.85 ± 0.51	9.53 ± 0.42	<0.0001
Glycolysis profile (mean ± SD)		10.48 ± 0.55	10.84 ± 0.46	10.63 ± 0.43	0.0073
Claudin-low	Claudin-low	0	1	9	0.0002
	Other	24	47	26	

BL, basal-like; CC, cell cycle; GES, gene-expression signatures; IM, immunomodulatory; IR, immune response; LAR, luminal androgen receptor; M, mesenchymal; MSL, mesenchymal stem-like; NBL, normal breast-like; SR, steroid hormone receptor; SSP, single sample predictor; VEGF, vascular endothelial growth factor.

**Single sample predictor annotation** Assignment of patients to a particular molecular subtype by means of SSP is dependent on the SSP used; for this reason, we used three available SSPs [27]. These signatures showed that C1 essentially contained non-basal-like subtypes (Table 3, Additional file 5). This cluster was mostly composed of luminal A and B subtypes, and unclassified tumours. In C1, PAM50 subtyping identified 79.1% of luminal subtypes (luminal A (20.8%) and B (58.3%)) and only one basal-like tumour. According to luminal enrichment of C1 and IHC results for AR, C1 should be named LAR. C2 was an almost pure basal-like cluster whatever the SSP used. In C2, one patient, who was subtyped as normal breast-like by means of PAM50, unclassified by Hu’s SSP and basal-like by Sorlie’s SSP, presented an adenoid cystic carcinoma, which is considered as a TN phenotype with an excellent prognosis [28]. This could explain why this tumour had a different molecular assignment compared to other C2 tumours. C3 included mostly basal-like subtypes, but to a lesser extent than C2 (91.4% by PAM50 SSP, 85.7% by Hu’s SSP and 65.7% by Sorlie’s SSP).

**Proliferation score** Proliferation score significantly decreased from C2 to C3 (*P* < 0.0074), then from C3 to C1 (*P* < 0.0001). It is important to notice that the two basal-like-enriched clusters (C2 and C3) presented a significantly different proliferation score.

**TNBCtype** TNBCtype classification assigned a TNBC subtype to 78.5% of our tumours (Table 3, Additional file 6). Most of the unstable (that is, unclassified) tumours belonged to C1 (48%). C1 was LAR-enriched (61.5% of classified patients), and LAR subtypes were exclusively assigned to this cluster. This result confirmed that C1 was not a basal-like cluster as shown with the SSPs. C2 was basal-like 1 enriched and mesenchymal enriched (47.6% and 42.8%, respectively). Basal-like 1 and basal-like 2 represented 55% of C2. C3 was immunomodulatory enriched and mesenchymal stem-like enriched (65.5% and 13.8%, respectively). Mesenchymal stem-like, characterized by low expression of proliferation genes compared to mesenchymal-like, was mostly found in C1 and C3. All immunomodulatory subtypes were included in C3. Immune response distinguished C3 from C2. Distribution of TNBCtype subtypes as a function of PAM50 basal-like or non-basal-like led to the same conclusion as Masuda and colleagues [29] - non-PAM50 basal-like subtypes (C1 in our study) were mostly composed of LAR and mesenchymal stem-like subtypes (unclassified excluded).

**Teschendorff’s gene-expression signature** Teschendorff’s GES has been designed for ER-negative tumour subtyping. Steroid hormone receptor, cell cycle (CC+) and cell cycle and immune response (CC+/IR+) subtypes were exclusively observed in C1, C2 and C2 + C3, respectively (Figure 3). Of note, most frequently immune response was mixed with cell cycle (CC+/IR+) (*n* = 74). Only two “pure” IR+ subtypes were found (one in C1 and one in C3). This GES confirmed TNBCtype subtyping of C1 as steroid hormone receptor and LAR. Immune response was almost exclusively assigned to C2 and C3 but did not separate these two clusters.

**Vascular endothelial growth factor profile** This 13-GES showed that angiogenesis varied according to the three clusters (*P* < 0.0001). Angiogenesis increased from C1 to C3 (*P* = 0.0274), and then from C3 to C2 (*P* = 0.0114).

**Glycolysis profile** Glycolysis score was significantly different according to cluster (*P* = 0.0073). The score of C1 was inferior to that of C2 (*P* = 0.0073) but no different from that of C3 (*P* = 0.44). A slight trend was found between C2 and C3 (*P* = 0.12).

**Signature correlation analyses** VEGF/glycolysis, proliferation/VEGF and proliferation/glycolysis profiles were positively correlated for patients included in our TN cohort (r = 0.62, *P* < 0.0001; r = 0.41, *P* < 0.0001; r = 0.48, *P* < 0.0001, respectively). Furthermore, correlation between VEGF and glycolysis profiles was found in each cluster (C1: r = 0.63, *P* = 0.0009; C2: r = 0.57, *P* < 0.0001; C3: r = 0.52, *P* = 0.0015). It is important to note that the gene lists for VEGF score, glycolysis score and proliferation score have no gene in common and, thus, correlations can be attributed to tumour biology.

**Claudin-low** Ten patients were subtyped as “claudin-low” (9.3%). The distribution was: 0% in C1, 2% (1/48) in C2 and 26% (9/35) in C3. Thus, C3 was claudin-low enriched but this subtype only represented a quarter of its number. Claudin-low compared to PAM50 basal-like tumours (*n* = 70) showed significant lower proliferation (*P* = 0.0076, Wilcoxon’s test). This result is in concordance with current knowledge. Prat and colleagues concluded that these tumours were likely slower-cycling tumours [6].

**Rody’s gene expression signature** In order to dissect immune response, we applied Rody’s immune metagenes to patients belonging to C2 and C3. In doing so, seven immune modules representing immune cells or immune processes were distinguished: B lymphocytes (IgG); macrophages and monocyte/myeloid lineage cells (HCK); professional antigen-presenting cells (MHC-II); T cells (LCK); cell types for presentation of intracellular antigens (MHC-I); interferon signal transduction (STAT1); and interferon response (interferon)) [15]. For each immune module, metagene expressions were always significantly higher in C3 compared to C2 (Additional file 7). Based on a variety of immune cells and immune processes, these results demonstrated that high immune response (HIR) could be considered as a hallmark of C3. Considering C2 and C3 patients, the impact of Rody’s metagenes on EFS and OS analyses was assessed. High expression of the majority of these metagenes was significantly associated with a better outcome: MHC-II (EFS: *P* = 0.0045, hazard ratio (HR)  = 0.61; OS: *P* = 0.0117, HR = 0.57), interferon (EFS: *P* = 0.007, HR = 0.66; OS: *P* = 0.0138, HR = 0.63), STAT1 (EFS: *P* = 0.004, HR = 0.68; OS: *P* = 0.0069, HR = 0.64), LCK (EFS: 0.0065, HR = 0.66; OS: *P* = 0.0086, HR = 0.60) and HCK (EFS: *P* = 0.0276, HR = 0.62; OS: *P* = 0.06). A trend was found for MHC-I (EFS: *P* = 0.08; OS: *P* = 0.14). IgG module was not associated with disease evolution (EFS: *P* = 0.69; OS: *P* = 0.43).

**Figure 4:**
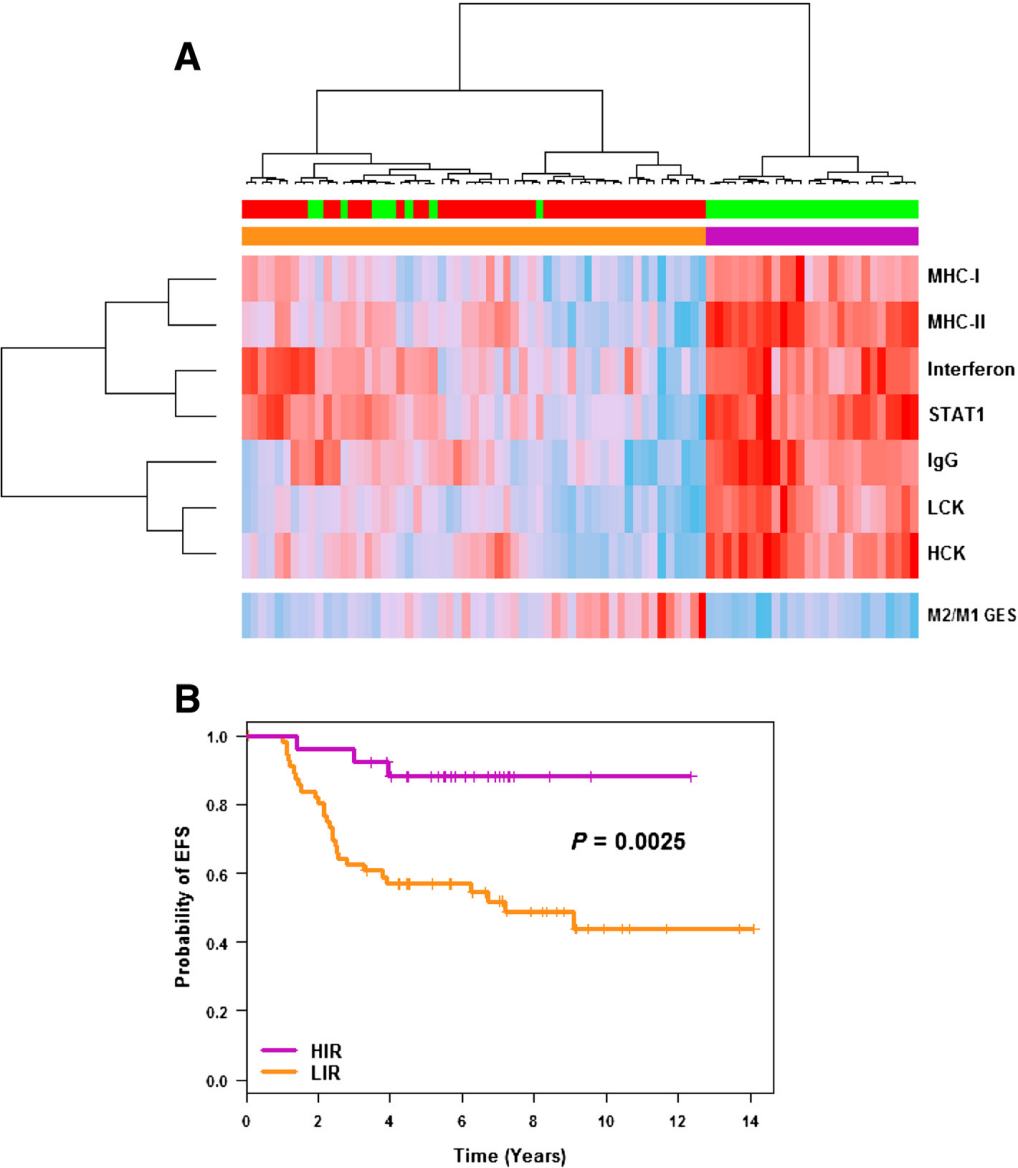
**C2/C3 immune response dissection. (A)** Ward’s hierarchical clustering and heatmap showing the segregation of C2 (red) and C3 (green) patients as a function of the seven Rody’s metagenes (B lymphocytes (IgG); macrophages and monocyte/myeloid lineage cells (HCK); professional antigen-presenting cells (MHC-II); T-cell (LCK); cell types for presentation of intracellular antigens (MHC-I); interferon signal transduction (STAT1); interferon response (interferon)) and M2/M1 gene-expression signature (GES) (continuous colour scale, from minimum (8.05, 6.90, 5.06, 7.84, 7.43, 9.68, 11.19 and −1.36 for HCK, LCK, IgG, STAT1, Interferon, MHC-II, MHC-I and M2/M1 GES, respectively; deep sky blue) to maximum (11.46, 12.24, 12.93, 13.21, 12.55, 13.75, 14.69 and −0.68 for HCK, LCK, IgG, STAT1, Interferon, MHC-II, MHC-I and M2/M1 GES, respectively; red). **(B)** Kaplan-Meier curves for event-free survival (EFS) analysis of breast cancer patients with high immune response (HIR) and low immune response (LIR).

Ward’s hierarchical clustering applied to these immune modules separated C2 and C3 patients into two clusters: one exclusively composed of C3 patients with HIR (*n* = 26) and the other composed of a majority (84%) of C2 patients with low immune response (LIR) (Figure 4A). All TNBCtype immunomodulatory (*n* = 19) were included in the HIR group. EFS analysis showed that HIR patients had a significantly better outcome than LIR patients (*P* = 0.0025; Figure 4B). Hence, immune response subtyping separated TN basal-like patients into two groups with different outcomes. A pooled cohort composed of C2, C3 and their equivalent in the external cohort, C2’ and C3’, (*n* = 141) confirmed this finding (*P* = 0.0212; *n* = 70 and 71, respectively for LIR and HIR) (Additional file 8). In our study, Rody’s metagenes were more powerful than Teschendordff’s GES to separate C2 and C3 as a function of immune response.

**M2/M1 GES** Of the 649 genes found differentially expressed by the SAM method, 611 were measured in Affymetrix® Human Genome U133 Plus 2.0 Arrays and used for macrophage subtyping (Additional file 9). Seventy-nine genes were in common between M2/M1 GES and the C2 versus C3 genes list, which included most differentially expressed genes between C2 and C3. Considering only C2 and C3 clusters, M2/M1 GES was significantly associated with a bad outcome (*P* = 0.02 and 0.04, respectively, for EFS and OS). M2 pro-tumourigenic macrophage genes were more expressed in C2 (*P* < 0.0001), which was characterized by LIR and bad outcome, and M1 tumour suppressor macrophage genes were more expressed in C3, which was characterized by HIR and better outcome. Of note, we did not find any correlation between VEGF score and M2 macrophage signature (r = 0.08; *P* = 0.45).

#### Single gene-expression intuitive approach

C1 was characterized by overexpression of luminal markers (*AR*, *ESR1*, *GATA3*, *KRT18*, *KRT19* and *MUC1*) and low expression of basal-like markers (*CDH3*, *KIT*, *KRT5*, *KRT6B*, *KRT14*, *KRT17*) compared to C2 and C3, leading us to name it LAR (Additional file 10). *ESR1* distribution according to the three clusters showed a significantly higher expression in C1 compared to C2 (*P* = 0.0022) and a trend with C3 (*P* = 0.07). *ERBB2* was only found overexpressed between C1 and C3. Higher expression of “epithelial cell-cell adhesion” (*CDH1* and *EPCAM*) and proliferation markers (*MKI67*, *UBE2C*, *RACGAP1*) was observed between C2 and C3. Low expression of EMT (*CDH2*, *TGFB1*), “immune system response” (*CD4*, *CD79A*, *CXCL2*, *IL6*, *VAV1*), breast stem cells (*ABCA8*, *ALDH1A1*) and angiogenesis markers (*TEK*, *TIE1*, *VEGFC*) in C2 differentiated this cluster from C3. In regard of these results, we may conclude that C2 is a true “basal-like” cluster. As detailed above, biological behaviour of C3 was different from C2. Most of the underlined differences, except the three claudin genes (*CDLN3*, *CDLN 4* and *CDLN7*), characterized a “claudin-low” cluster that expressed low luminal differentiation markers, high EMT markers, immune response genes, cancer stem cell-like features and a stem cell-associated biological process. In regard to a preponderance of immune response genes in C3, we thought that “immune-related” was more suited to this last cluster.

#### Gene ontology biological process terms enrichment

One overexpressed gene probe cluster was identified for each cluster of patients by means of the 5% most variant gene probes (*n* = 2,734) heatmap. Those clusters were named H1 for C1, H2 for C2 and H3 for C3. H1, H2 and H3 were composed of 481, 442 and 222 gene probes, respectively (Additional file 11). Lists of discriminating gene probes (C1 versus C2, C1 versus C3, C2 versus C3 and C1 versus C2 and C3) were composed of 682, 575, 565 and 565 gene probes, respectively. Twenty-one GO biological process terms enrichment analyses demonstrated that C1 was characterized by epithelial cell differentiation and a hormone metabolic process, C2 by cell adhesion, locomotion and chemotaxis, and C3 by immune response (Additional file 12).

### External validation

**Figure 5:**
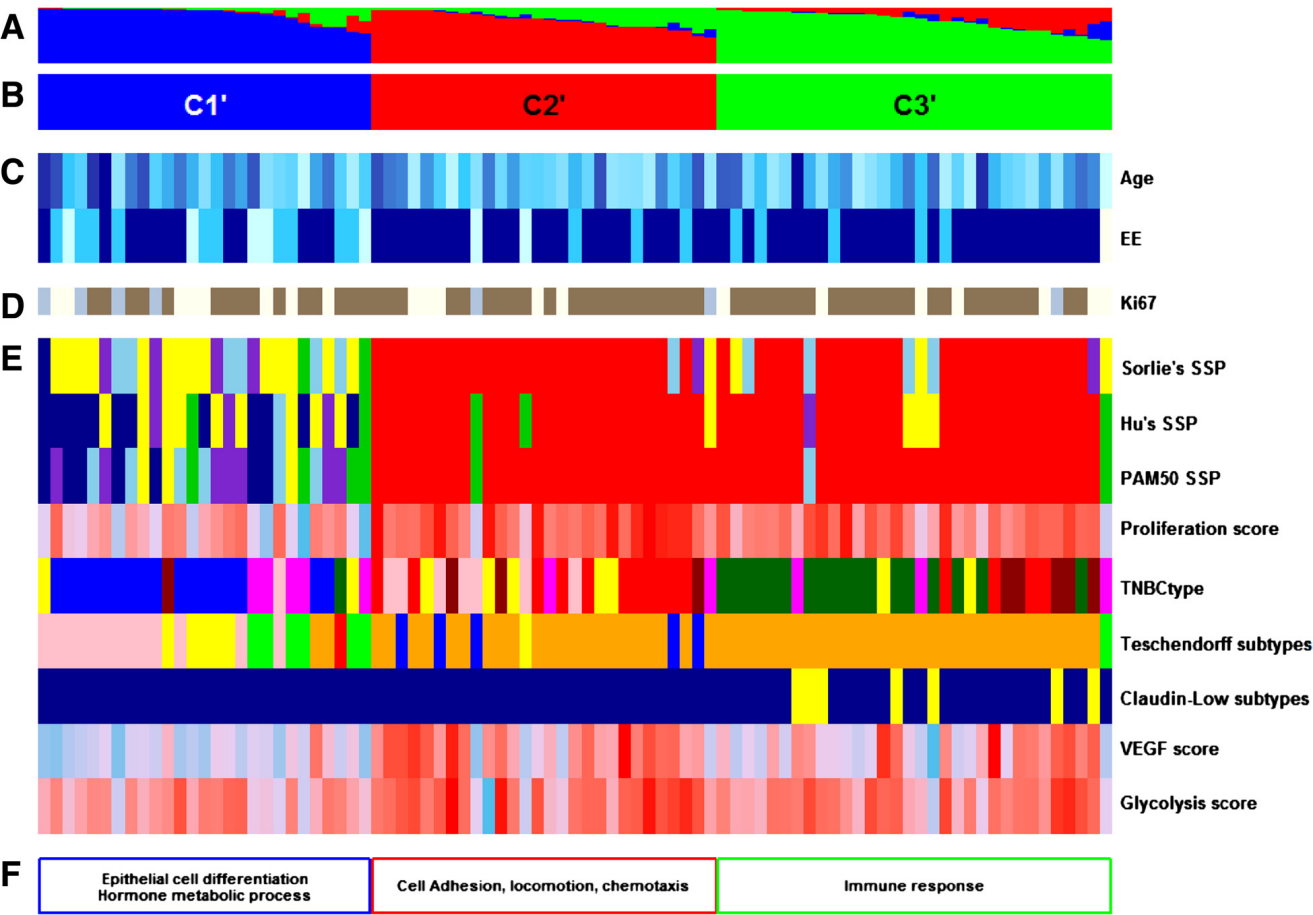
**Functional annotations of the clustering results for triple-negative patients from GSE21653 (** ***n*** **= 87). (A)** Fuzzy-clustering probability of belonging to clusters, from 0 to 1. **(B)** Cluster numbering scheme. **(C)** Clinicopathologic characteristics with significant differences between clusters: age as a continuous colour scale, from 28 years old (pale turquoise) to 85 years old (dark blue); Elston-Ellis (EE) histological grades 1 (pale turquoise), 2 (deep sky blue), and 3 (dark blue); Nottingham prognostic index (NPI) 1 (pale turquoise), 2 (deep sky blue), and 3 (dark blue). **(D)** Immunohistochemistry results for Ki67: positive (brown) and negative (blue). **(E)** Molecular subtyping by means of nine gene expression signatures (GES): three single sample predictors (SSPs) - luminal A (dark blue), luminal B (sky blue), HER2-E (purple), basal-like (red), normal breast-like (green) or unclassified (yellow); proliferation (continuous colour scale from minimum (6.01; deep sky blue) to maximum (10.92; red)); TNBCtype (basal-like 1 (red), basal-like 2 (dark red), immunomodulatory (dark green), mesenchymal-like (pink), mesenchymal stem-like (magenta), luminal androgen receptor (blue) and unclassified tumours (yellow)); Teschendorff’s GES (cell cycle (blue), cell cycle and immune response (orange), extracellular matrix (green), immune response (red), steroid hormone response (pink) and unclassified (yellow)); claudin-low (claudin-low (yellow), other (dark blue)); vascular endothelial growth factor (VEGF; continuous colour scale, from minimum (8.12; deep sky blue) to maximum (10.82; red)); and glycolysis (continuous colour scale, from minimum (9.42; deep sky blue) to maximum (13.00; red)). **(F)** Gene ontology biological process terms enrichment. Missing values are in white.

Eighty-seven TN patients from GSE21653 were used for external validation. The same global process, as described for our cohort, was used. Results are summarized in Figure 5 and detailed in Additional file 13. In accordance with the clustering results of our cohort, GSE21653 TN patients were divided up into one non-basal-like cluster (C1’) and two basal-like clusters (one named true basal-like or basal-like (C2’) and the other, which included claudin-low subtypes (28%), characterized by immune response (C3’)). One difference was that the GSE21653 cohort included PAM50 HER2-E subtypes in C1’. Moreover, only five patients (5.74%) were differently classified with PAM.

## Discussion

Our work has once again raised the question of finding a gold standard method for breast cancer molecular subtyping; personalized medicine cannot do without effective identification of patients. We conducted an unsupervised analysis of microarray gene-expression profiles of a TN cohort composed of 107 patients. The robustness of our results was based on concordance of numerous and complementary functional annotation means: bio-clinical data (age, histological grade, NPI score), immunohistochemistry markers (CK5, HER1, Ki67, AR and FOXA1), 17 GES, intuitive single gene-expression approach and GO enrichment. Furthermore, our analysis was reinforced by external validation which confirmed our results. Functional annotation of clusters obtained by means of fuzzy clustering showed that 22% of these patients were not basal-like (C1 = LAR), which is in concordance with other works [30]. Of note, in GSE21653, 20 to 30% of SSP classified C1’ patients were subtyped as HER2-E; *ERBB2* expression was in concordance with this result (Additional file 13). As a consequence of this, the TN C1 cluster should be named “non-basal-like”, because it might be composed of non-basal-like patients with LAR and/or HER2-E characteristics. The two other clusters were enriched in basal-like subtype: C2, which could be considered as an almost pure basal-like cluster, included patients with bad outcome, and C3, enriched in basal-like subtypes but to a lesser extent, included patients with better outcome. C3 was composed of 26% claudin-low subtypes. Of note, claudin-low patients were subtyped by means of GES. Our cohort was probably too small to individualize a well-separated claudin-low subtype; Prat and colleagues initially characterized claudin-low subtype in a cohort of 337 patients [6]. Dissection of immune response, which was high in these two last clusters compared to C1, was first performed by means of seven metagenes. Results showed that HIR was a hallmark of C3, and that HIR was associated with a better outcome than LIR (C2). This was true for our cohort (*P* = 0.0195) alone and pooled with GSE21653 TN patients (*P* = 0.002) (Figure 2, Additional file 4). We proposed that C3 should be called “HIR”, because HIR had the clinical advantage of individualizing a cluster of patients with good outcome. C2 seemed to correspond to “basal-like immune-suppressed”, and C3 to “basal-like immune-activated”, recently described by Burstein and colleagues [31].

In addition to other studies, our work demonstrated that CK5 should be used rather than CK5/6 to identify basal-like subtype. Indeed, in our cohort, sensitivity (based on C2 and C3 results) was 83% for CK5 and only 34% for CK5/6; both markers had similar specificity (based on C1 results): 77% for CK5 and 82% for CK5/6. In C1, 5 cases out of 22 were positive for CK5 (23%), 4/22 were positive for CK5/6 (18%) and 11/22 were positive for HER1 (50%). In 2004, Abd El-Rehim and colleagues showed that 27.4% of breast tumours displayed a combined luminal and basal phenotype (positivity for one or more of the luminal markers together with one or more of the basal markers) [32]. This could explain why CK5- or CK5/6-positive tumours were found in C1.

We focused on immune response, which is known to play a major role in tumour progression, because it was the main characteristic allowing us to distinguish between the two basal-like-enriched clusters: C2 (LIR) and C3 (HIR). Furthermore, actors or mechanisms of immune networks constitute potential drug targets suggesting that some TN patients might benefit from immune-based therapies. In cancer, some immune cells are known to induce anti-tumoural effects (natural killer cells, CD8+ T cells, Th1 cells, dendritic cells 1, M1 macrophages, and so forth), and others induce pro-tumoural effects (myeloid-derived suppressor cells, CD4+ T cells, Th2 cells, dendritic cells 2, M2 macrophages, and so forth) [33,34]. These effects result from complex cross talk between immune cells, tumour cells and other cell populations of the microenvironment by means of extracellular signals, including many cytokines and their soluble receptors [35,36]. In breast cancer, recent works confirmed that tumour infiltrating lymphocytes (TILs) were most frequently found in highly proliferative tumours, including TN, and were associated with a favourable outcome [37,38]. Today, TILs are becoming a new breast cancer marker, particularly in TN and HER2-positive breast cancer [33]. As recommended by an international working group of experts in 2014, TIL score must take into account all mononuclear cells including lymphocytes and plasma cells, and excludes granulocytes, other polymorphonuclear leukocytes, dendritic cells and macrophages [33]. Nevertheless, macrophages are key modulator and effector cells in immune response and tumour progression [35]. An old, but still used, classification of macrophages roughly separates them into two “extreme” polarized subpopulations: the classically activated type 1 macrophages (M1) and the alternative activated type 2 macrophages (M2). In 2010, Qian and Pollard described six macrophage functions (activated, immunosuppressive, angiogenic, metastasis-associated, perivascular and invasive) ascribed to a unique macrophage subpopulation [39]. Recently, a working group of macrophage biologists defined a nomenclature and experimental guidelines to take into account the heterogeneity of macrophage activation and polarization [40]. They recommended a nomenclature linked to the activation standards and counted seven human macrophage subpopulations, three M1 types and four M2 types [40]. Beside the extreme phenotypes, intermediate phenotypes of macrophages should also be evaluated when dissecting immune response in cancer [41,42]. Within this cell group, tumour-associated macrophages (TAMs), which are considered as M2-like, represent a major source of proteases involved in tumour progression [43]. Very synthetically, polarized macrophages, together with fibroblasts and vascular endothelial cells from the tumour microenvironment, in conjunction with tumour cells, intervene at different stages of the cancer process. They have been implicated in the angiogenic switch (high production of VEGF), local invasion, and metastasis. Thus it is important to take into account these cells when dissecting immune response, and to identify their subpopulations because they have negative and positive effects on cancer evolution.

In this work, HIR, objectivised by means of Rody’s and TNBCtype GES, was exclusively related to “anti-tumoural” response; none of these GES were related to “pro-tumourigenic” response. Furthermore, Teschnedorff’s GES was unable to dissect immune response; immune response was high in C2 and C3 (Figure 3E). Almost all Rody’s immune modules showed that high expression was linked to good outcome (Figure 4). Expression of LCK and IgG Rody’s metagenes, as markers for T and B cells, were in concordance with current knowledge. On the contrary, high expression of HCK, which take into account macrophages and monocyte/myeloid lineage cells, was associated with favourable outcome. This result was a bit surprising because, in breast cancer, TAMs are known to be associated with bad prognosis [34]. Our hypothesis was that HCK was unable to distinguish between the two extreme macrophage phenotypes. For this reason, we established a M2/M1 GES to subtype TAMs into M1-like or M2-like groups. According to new nomenclature, M2/M1 GES, which should be named M(IL-4)/M(IFN-γ) GES, showed that M(IL-4) (M2 pro-tumourigenic macrophages) were more frequent in LIR (C2), which was characterized by a bad outcome, and that M(IFN-γ) (M1 tumour suppressor macrophages) were more frequent in HIR (C3), which was characterized by a favourable outcome. In other words, C2 was characterized by high pro-tumourigenic and low anti-tumoural immune response and C3 by low pro-tumourigenic and high anti-tumoural immune response. Finally, these results are in concordance with current knowledge, and are of particular importance because M2 inhibition, which may play a major pro-tumourigenic role in C2, represents a potential therapeutic strategy. Repolarization of M2 into M1 macrophages and inhibition of M2 macrophages both represent promising ways to treat cancer [44,45]. Cancer treatment may take advantage of therapies that interfere with M2 macrophages. Although the following model was not based on breast cancer, but on nude mice xenografted with colon cancer cells, GTP cyclohydrolase (CGH1) inhibition by 2,4-diamino-6-hydroxypyrimidine reduced tumour growth and angiogenesis by shifting the phenotype of tumour-associated macrophages from pro-angiogenic M2 towards M1 [46].

However, some caution must be taken with our results. First, our main hypothesis was that immune cells and particularly macrophage subpopulations represented main key effectors of TN breast cancer. Today, numerous concordant studies support this hypothesis [47-51]. Second, we showed that the M2 macrophage gene-expression profile was associated with bad outcome, and the reverse for M1. At this step, we did not demonstrate a causal link between M2 macrophage cells and outcome. Further analyses are required to dissect M2-dependent causal chains or systems that might explain breast cancer progression and evolution. Phenotypic markers should also be tested to validate macrophage distribution. Third, new TN patients are needed to test the strength of this partition.

## Conclusions

We identified three subtypes of TN patients: 1) non-basal-like (22%); 2) basal-like with LIR and high M2-like macrophages (45%); and 3) basal-enriched, including claudin-low subtypes, with HIR and low M2-like macrophages (33%). Our study added another well-defined TN cohort to the scientific community. We showed that our gene-expression data could be pooled with others to strengthen unsupervised analyses and to feed databases [52]. As others have done, we concluded that around 25% of TN patients, including non-basal-like cluster (LAR in our study), could receive hormonotherapy or anti-HER2 therapy. Furthermore, based on concordance between robust molecular results and IHC, we showed that the CK5 antibody was better suited than the CK5/6 antibody to subtype basal-like patients. This result is of importance within the framework of the search for a TN subtyping gold standard method. In this work, we pointed out that macrophages, particularly M2-like, offered a variety of therapeutic targets in TN patients. In breast cancer, and particularly in cases of TN basal-like tumours, future clinical trials evaluating novel immune therapies will have to be set up with patients stratified by means of the composition of their leukocyte infiltrate, which should include macrophage subpopulation identification [53]. A lot of work needs to be done in order to define this new multiparametric biomarker panel. To this end, cytology together with expression microarray data deconvolution seem more likely to succeed [54,55]. To conclude, we hypothesize that TN basal-like treatment, among other things, will have to restore the normal balance of immune cells, by means of targeted immune cell inhibition and/or augmentation, and that immune therapy will become an important component of TN basal-like combined therapies.

## Additional files

Additional file 1:
**Details of antibodies used for immunohistochemistry.**

Additional file 2:
**Interpretation of immunohistochemical staining.**

Additional file 3:
**Gene-expression signatures (GES) used for fuzzy cluster functional annotation.**

Additional file 4:
**Kaplan-Meier curves for event-free survival analysis based on fuzzy-clustering partition.** **(A)** GSE21653 triple negative (TN) breast cancer patients (*n* = 87). **(B)** Kaplan-Meier analysis of pooled cohorts (ours and GSE21653; *n* = 194) shows significantly increased event-free survival in C3 patients compared to C1 (*P* = 0.03) and C2 patients (*P* = 0.002).
Additional file 5:
**Distribution of single sample predictor (SSP) subtypes according to the three clusters of our cohort.** **(A)** Sorlie’s SSP. **(B)** Hu’s SSP. **(C)** Parker’s SSP (PAM50). Bar graph plots shows the distribution of intrinsic subtypes: luminal A (dark blue) and B (light blue), HER2-E (purple), basal-like (red), normal breast-like (green) or unclassified (yellow) tumours within each cluster. Whatever the SSP, subtype distributions show that C1 is a luminal-enriched cluster and not a basal-like cluster, C2 is an almost pure basal-like cluster, and C3 is a basal-like-enriched cluster.
Additional file 6:
**Distribution of TNBCtype subtypes according to the three clusters of our cohort.** Bar graph plots show the distribution of the six TNBCtype subtypes: basal-like 1 (red), basal-like 2 (dark red), immunomodulatory (dark green), mesenchymal-like (pink), mesenchymal stem-like (magenta), luminal androgen receptor (blue) and unclassified tumours (yellow), within each cluster.
Additional file 7:
**Rody’s metagene expression levels according to our cohort’s clusters.** For each immune module, *P* value of Tukey's test for the comparison between C2 and C3 is indicated. Metagene expressions are always significantly higher in C3 compared to C2.
Additional file 8:
**Kaplan-Meier curves for event-free survival analysis based on Rody’s metagenes clustering.** Rody’s metagenes clustering was based on patients included in C2 and C3 (our cohort; see Figure 4A), and in C2’ and C3’ (GSE21653) (low immune response (LIR), *n* = 70; high immune response (HIR), *n* = 71).
Additional file 9:
**M2/M1 gene expression signature (GES) genes list.** List of the 611 genes used to compute the M2/M1 GES and their relative expression in M2 versus M1 population.
Additional file 10:
**Expression level of chosen genes between the three fuzzy clusters of our cohort.**

Additional file 11:
**Fuzzy clustering heatmap of the 5% most variant gene probes.** Each column represents a patient (*n* = 107) and each row an Affymetrix gene probe (*n* = 2,734). Green indicates low expression, red indicates high expression. Clusters of overexpressed genes used for gene ontology (GO) biological process terms enrichment are indicated by a bracket: H1 for C1 (blue), H2 for C2 (red) and H3 for C3 (green). H1, H2 and H3 were composed of 481, 442 and 222 gene probes, respectively.
Additional file 12:
**Biological process gene ontology (GO) terms enrichment for our cohort.**

Additional file 13:
**External validation process results.** Eighty-seven triple negative (TN) patients of GSE21653 cohort were used for external validation. The same global process, as described for our cohort, was used and is described thereafter.

## Abbreviations

AR: androgen receptor
EFS: event-free survival
EMT: epithelial-to-mesenchymal transition
ER: oestrogen receptor
GEO: Gene Expression Omnibus
GEP: gene-expression profiling
GES: gene-expression signatures
GO: gene ontology
IHC: immunohistochemistry
HER2-E: HER2-enriched
HES: hemalun-eosin-safran
HIR: high immune response
HR: hazard ratio
LAR: luminal androgen receptor
LIR: low immune response
NPI: Nottingham prognostic index
OS: overall survival
PAM: prediction analysis for microarrays
PR: progesterone receptor
SAM: significance analysis of microarrays
SBR: Scarff Bloom Richardson
SSP: single sample predictor
TAM: tumour-associated macrophage
TIL: tumour infiltrating lymphocyte
TN: triple-negative
TNBCtype: a subtyping tool for triple-negative breast cancer
VEGF: vascular endothelial growth factor
