# Supplementary material for: Gene-expression molecular subtyping of triple-negative breast cancer tumours: importance of immune response
Source: Breast Cancer Res. 2015 Mar 20;17:43. doi: 10.1186/s13058-015-0550-y (PMC4389408; doi:10.1186/s13058-015-0550-y)
Supplement: Additional file 1: — Details of antibodies used for immunohistochemistry. [file 13058_2015_550_MOESM1_ESM.pdf]

Additional file 1: Details of antibodies used for immunohistochemistry.

**Antibody**	**Clone**	**Dilution**	**Antigen retrieval**	**Supplier**
CK5/6	D5/16B4	1/50	EDTA pH9	Dako
CK5	XM26	1/50	Citrate buffer pH6	Dako
HER1	31G7	1/20	Citrate buffer pH6	Zymed
AR	AR441	1/75	Citrate buffer pH6	Dako
Ki-67	MIB1	1/150	Citrate buffer pH6	Dako
FOXA1	Polyclonal	1/100	Citrate buffer pH6	Diagomics
E-cadherin	NCH38	1/100	Citrate buffer pH6	Dako
Claudin 3	Polyclonal	1/100	Citrate buffer pH6	Diagomics
Claudin 4	Polyclonal	1/100	Citrate buffer pH6	Diagomics
Claudin 7	Polyclonal	1/100	Citrate buffer pH6	Diagomics
