# Supplementary material for: Gene-expression molecular subtyping of triple-negative breast cancer tumours: importance of immune response
Source: Breast Cancer Res. 2015 Mar 20;17:43. doi: 10.1186/s13058-015-0550-y (PMC4389408; doi:10.1186/s13058-015-0550-y)
Supplement: Additional file 2: — Interpretation of immunohistochemical staining. [file 13058_2015_550_MOESM2_ESM.pdf]

Additional file 2: Interpretation of immunohistochemical staining.

Marker	IHC staining	Cut-off, score	Interpretation	Reference
CK5/6	cytoplasmic	≥ 1%	Positive	[1]
CK5	cytoplasmic	≥ 1%	Positive	[2]
HER1	membrane	≥ 1%	Positive	[3]
AR	nuclear	< 10%	Negative	[4]
		≥ 10%	Positive	
Ki-67	nuclear	< 14%	Low proliferation	[5]
		≥ 14%	High proliferation	
FOXA1	nuclear	0 to 3	Low	[6]
		4 to 30	High	
E-cadherin	membrane	0 to 1	Negative	[7]
		2 to 3	Positive	
Claudin 3, 4 and 7	membrane	0 to 2	Low	[8]
		3 to 9	High	

References

1. Nielsen TO, Hsu FD, Jensen K, Cheang M, Karaca G, Hu Z, Hernandez-Boussard T, Livasy C, Cowan D, Dressler L, Akslen LA, Ragaz J, Gown AM, Gilks CB, van de Rijn M, Perou CM: **Immunohistochemical and clinical characterization of the basal-like subtype of invasive breats carcinoma.** *Clin Cancer Res* 2004, **10:**5367-5374.
2. Alshareeda AT, Soria D, Garibaldi JM, Rakha E, Nolan C, Ellis IO, Green AR: **Characteristics of basal cytokeratin expression in breast cancer.** *Breast Cancer Rest Treat* 2013,**139:**23-37.
3. Gori S, Sidoni A, Colozza M, Ferri I, Mameli MG, Fenocchio D, Stocchi L, Foglietta J, Ludovini V, Minenza E, De Angelis V, Crinò L: **EGFR, pMAPK, pAkt and PTEN status by immunohistochemistry: correlation with clinical outcome in HER2-positive metastatic breast cancer patients treated with trastuzumab.** *Ann Oncol* 2009, **20:**648-654.
4. Park S, Koo J, Park HS, Kim JH, Choi SY, Lee JH, Park BW, Lee KS: **Expression of androgen receptors in primary breast cancer.** *Ann Oncol* 2010, **21:**488-492.
5. Goldhirsch A, Wood WC, Coates AS, Gelber RD, Thürlimann B, Senn HJ: **Strategies for subtypes-dealing with the diversity of breast cancer: highlights of the St Gallen international expert consensus on the primary therapy of early breast cancer 2011.** *Ann Oncol* 2011, **22:**1736-1747.
6. Thorat MA, Marchio C, Morimiya A, Savage K, Nakshatri H, Reis-Filho JS, Badve S: **Forkhead box A1 expression in breast cancer is associated with luminal subtype and good prognosis.** *J Clin Pathol* 2008, **61:**327-332.
7. Kashiwagi S, Yashiro M, Takashima T, Nomura S, Noda S, Kawajiri H, Ishikawa T, Wakasa K, Hirakawa K: **Significance of E-cadherin expression in triple-negative breast cancer.** *Br J Cancer* 2010, **103:**249-255.
8. Lu S, Singh K, Mangray S, Tavares R, Noble L, Resnick MB, Yakirevich E: **Claudin expression in high-grade invasive ductal carcinoma of the breast: correlation with the molecular subtype.** *Mod Pathol* 2013, **26:**485-495.
