# Supplementary material for: Gene-expression molecular subtyping of triple-negative breast cancer tumours: importance of immune response
Source: Breast Cancer Res. 2015 Mar 20;17:43. doi: 10.1186/s13058-015-0550-y (PMC4389408; doi:10.1186/s13058-015-0550-y)
Supplement: Additional file 3: — Gene-expression signatures (GES) used for fuzzy cluster functional annotation. [file 13058_2015_550_MOESM3_ESM.pdf]

Additional file 3: Gene-expression signatures (GES) used for fuzzy cluster functional annotation.

GES name	N° genes	Reference	Statistics	Subtypes or score assignment
Sorlie's SSP	500	[1]	Nearest centroid classifier	Basal-like
Hu's SSP	306	[2]		HER2-E
Parker's SSP = PAM50	50	[3]		Luminal A
				Luminal B
				Normal breast-like
Proliferation score	72	[4]	Average expression	Continuous score
TNBCtype	2188	[5]	Nearest centroid classifier	Basal-like 1 (BL1)
				Basal-like 2 (BL2)
				Immunomodulatory (IM)
				Luminal androgen receptor (LAR)
				Mesenchymal-like (M)
				Mesenchymal stem-like (MSL)
Teschendorff GES	813	[6]	Nearest centroid classifier	Cell cycle (CC)
				Cell cycle and immune response (CC+IR)
				Extracellular matrix (ECM)
				Immune response (IR)
				Steroid hormone response (SR)
VEGF profile	13	[7]	Average expression	Continuous score
Glycolysis profile	6	[7]	Average expression	Continuous score
Claudin-low	771	[8]	Nearest centroid classifier	Claudin-low
				No claudin-low
Rody's metagenes	153	[9]	Average expression	B lymphocytes (IgG)
				Cell types for presentation of intracellular antigens (MHC-I)
				Interferon response (Interferon)
				Interferon signal transduction (STAT1)
				Macrophages, monocyte/myeloid lineage cells (HCK)
				Professional antigen-presenting cells (MHC-II)
				T-cell (LCK)
M2/M1 GES	611	Our study	Weighted average expression	Continuous score (low score: M(IFN-γ) = macrophages (M1); high score: M(IL-4) = macrophages (M2))

SSP, single sample predictor; GES, gene-expression signature

References

[1] Sorlie T, Tibshirani R, Parker J, Hasties T, Marron JS, Nobel A, Deng S, Johnsen H, Pesich R, Geisler S, Demeter J, Perou CM, Lonning PE, Brown PO, Borresen-Dale AL, Botstein D: **Repeated observation of breast tumor subtypes in independent gene expression data sets.** *Proc Natl Acad Sci USA* 2003, **100:**8418-8423.

[2] Hu Z, Fan C, Oh DS, Marron JS, He X, Qaqish BF, Livasy C, Carey LA, Reynolds E, Dressler L, Nobel A, Parker J, Ewend MG, Sawyer LR, Wu J, Liu Y, Nanda R, Tretiakova M, Ruiz Orrico A, Dreher D, Palazzo JP, Perreard L, Nelson E, Mone M, Hansen H, Mullins M, Quackenbush JF, Ellis MJ, Olopade OI, Bernard PS, Perou CM: **The molecular portraits of breast tumors are conserved across microarray platform.** *BMC Genomics* 2006, **7:**96.

[3] Parker JS, Mullins M, Cheang MC, Leung S, Voduc D, Vickery T, Davies S, Fauron C, He X, Hu Z, Quanckenbush JF, Stijleman IJ, Palazzo J, Marron JS, Nobel AB, Mardis E, Nielsen TO, Ellis MJ, Perou CM, Bernard PS: **Supervised risk predictor of breast cancer based on intrinsic subtypes.** *J Clin Oncol* 2009, **27:**1160-1167.

[4] Dexter TJ, Sims D, Mitsopoulos C, Mackay A, Grigoriadis A, Ahmad AS, Zvelebil: **Genomic distance entrained clustering and regression modelling highlights interacting genomic regions contributing to proliferation in breast cancer.** *BMC Syst Biol* 2010, **4:**127.

[5] Chen X, Li J, Gray WH, Lehmann BD, Bauer JA, Shyr Y, Pietenpol JA: **TNBCtype: A subtyping tool for triple-negative breast cancer.** *Cancer Inform* 2012, **11:**147-156.

[6] Teschendorff AE, Miremadi A, Pinder SE, Ellis IO, Caldas C: **An immune response module identifies a good prognosis subtype in estrogen negative breast cancer.** *Genome Biol* 2007, **8:**R157.

[7] Hu Z, Fan C, Livasy C, He X, Oh DS, Ewend MG, Carey LA, Subramanian S, West R, Ikpatt F, Olopade OI, van de Rijn M, Perou CM: **A compact VEGF signature associated with distant metastases and poor outcomes.** *BMC Med* 2009, **7:**9.

[8] Prat A, Parker JS, Karginova O, Fan C, Livasy C, Herschkowitz JI, He X, Perou CM: **Phenotypic and molecular characterization of the claudin-low intrinsic subtype of breast cancer.** *Breast Cancer Res* 2010, **12:**R68.

[9] Rody A, Holtrich U, Pusztai L, Liedtke C, Gaetje R, Ruckhaeberle E, Solbach C, Hanker L, Ahr A, Metzler D, Engels K, Karn T, Kaufmann M: **T-cell metagene predicts a favorable prognosis in estrogen receptor-negative and HER2-positive breast cancers.** *Breast Cancer Res* 2009, **11:**R15.
