# Supplementary material for: Gene-expression molecular subtyping of triple-negative breast cancer tumours: importance of immune response
Source: Breast Cancer Res. 2015 Mar 20;17:43. doi: 10.1186/s13058-015-0550-y (PMC4389408; doi:10.1186/s13058-015-0550-y)

Additional file 4: Kaplan-Meier curves for event-free survival analysis based on fuzzy-clustering partition. (A) GSE21653 TN breast cancer patients (*n* = 87). **(B)** Kaplan-Meier analysis of pooled cohorts (ours and GSE21653; *n* = 194) shows significantly increased event-free survival in C3 patients compared to C1 (*P* = 0.03) and C2 patients (*P* = 0.002).

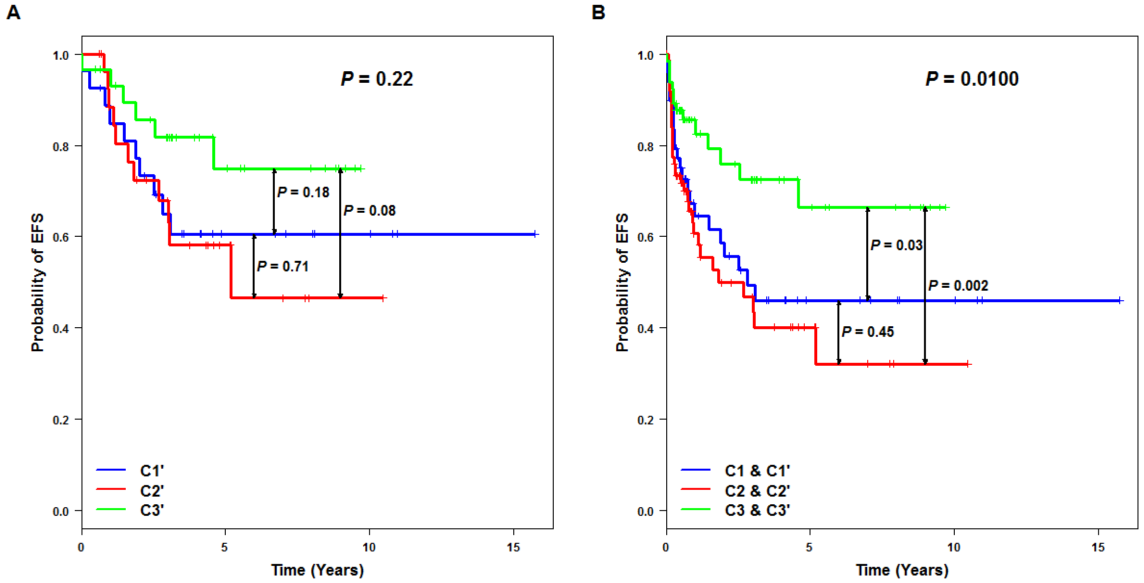

Supplement: Additional file 4: — Kaplan-Meier curves for event-free survival analysis based on fuzzy-clustering partition. (A) GSE21653 triple negative (TN) breast cancer patients (n = 87). (B) Kaplan-Meier analysis of pooled cohorts (ours and GSE21653; n = 194) shows significantly increased event-free survival in C3 patients compared to C1 (P = 0.03) and C2 patients (P = 0.002). [file 13058_2015_550_MOESM4_ESM.pdf]
