# Supplementary material for: Gene-expression molecular subtyping of triple-negative breast cancer tumours: importance of immune response
Source: Breast Cancer Res. 2015 Mar 20;17:43. doi: 10.1186/s13058-015-0550-y (PMC4389408; doi:10.1186/s13058-015-0550-y)

Additional file 5: Distribution of SSP subtypes according to the three clusters of our cohort. (A) Sorlie's SSP. **(B)** Hu's SSP. **(C)** Parker's SSP (PAM50). Bar graph plots shows the distribution of intrinsic subtypes: luminal A (dark blue) and B (light blue), HER2-E (purple), basal-like (red), normal breast-like (green) or unclassified (yellow) tumours within each cluster. Whatever the SSP, subtype distributions show that C1 is a luminal-enriched cluster and not a basal-like cluster, C2 is an almost pure basal-like cluster and C3 is a basal-like-enriched cluster.

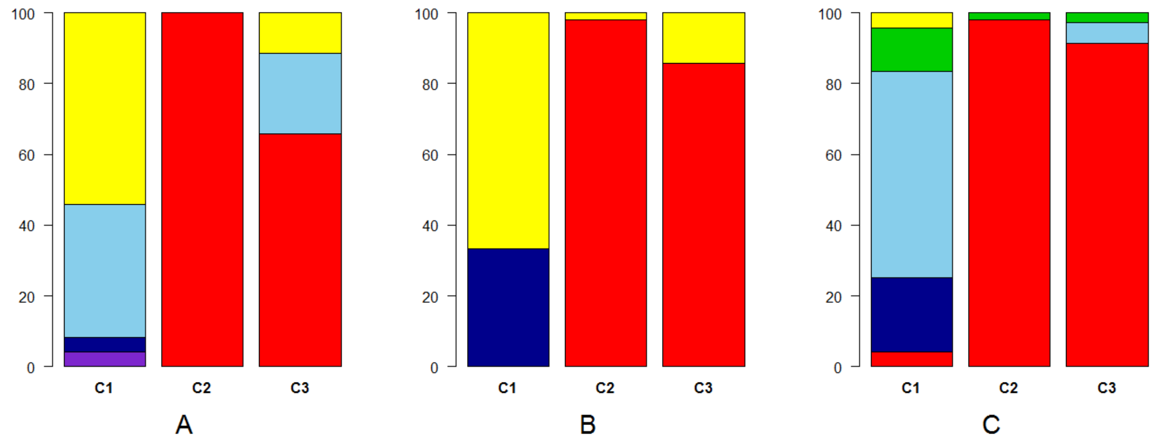

Supplement: Additional file 5: — Distribution of single sample predictor (SSP) subtypes according to the three clusters of our cohort. (A) Sorlie’s SSP. (B) Hu’s SSP. (C) Parker’s SSP (PAM50). Bar graph plots shows the distribution of intrinsic subtypes: luminal A (dark blue) and B (light blue), HER2-E (purple), basal-like (red), normal breast-like (green) or unclassified (yellow) tumours within each cluster. Whatever the SSP, subtype distributions show that C1 is a luminal-enriched cluster and not a basal-like cluster, C2 is an almost pure basal-like cluster, and C3 is a basal-like-enriched cluster. [file 13058_2015_550_MOESM5_ESM.pdf]
