# Supplementary material for: Gene-expression molecular subtyping of triple-negative breast cancer tumours: importance of immune response
Source: Breast Cancer Res. 2015 Mar 20;17:43. doi: 10.1186/s13058-015-0550-y (PMC4389408; doi:10.1186/s13058-015-0550-y)

Additional file 6: Distribution of TNBCtype subtypes according to the three clusters of our cohort. Bar graph plots show the distribution of the six TNBCtype subtypes: basal-like 1 (red), basal-like 2 (dark red), immunomodulatory (dark green), mesenchymal-like (pink), mesenchymal stem-like (magenta), luminal androgen receptor (blue) and unclassified tumours (yellow), within each cluster.

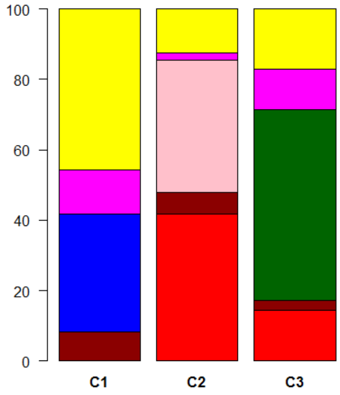

Supplement: Additional file 6: — Distribution of TNBCtype subtypes according to the three clusters of our cohort. Bar graph plots show the distribution of the six TNBCtype subtypes: basal-like 1 (red), basal-like 2 (dark red), immunomodulatory (dark green), mesenchymal-like (pink), mesenchymal stem-like (magenta), luminal androgen receptor (blue) and unclassified tumours (yellow), within each cluster. [file 13058_2015_550_MOESM6_ESM.pdf]
