# Supplementary material for: Gene-expression molecular subtyping of triple-negative breast cancer tumours: importance of immune response
Source: Breast Cancer Res. 2015 Mar 20;17:43. doi: 10.1186/s13058-015-0550-y (PMC4389408; doi:10.1186/s13058-015-0550-y)

Additional file 7: Rody's metagene expression levels according to our cohort's clusters. For each immune module, *P*-value of Tukey's test for the comparison between C2 and C3 is indicated. Metagenes expressions are always significantly higher in C3 compared to C2.

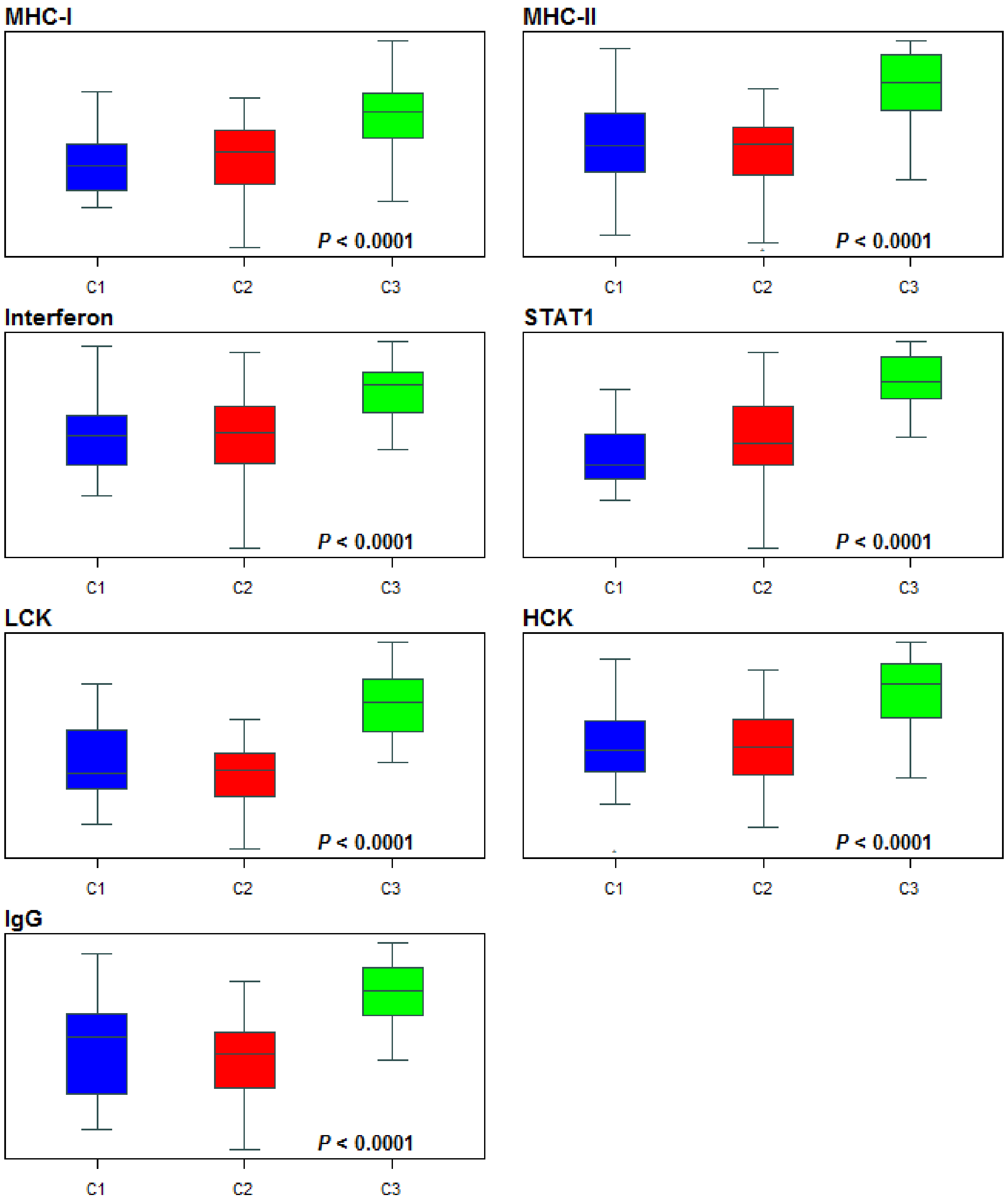

Supplement: Additional file 7: — Rody’s metagene expression levels according to our cohort’s clusters. For each immune module, P value of Tukey's test for the comparison between C2 and C3 is indicated. Metagene expressions are always significantly higher in C3 compared to C2. [file 13058_2015_550_MOESM7_ESM.pdf]
