# Supplementary material for: Gene-expression molecular subtyping of triple-negative breast cancer tumours: importance of immune response
Source: Breast Cancer Res. 2015 Mar 20;17:43. doi: 10.1186/s13058-015-0550-y (PMC4389408; doi:10.1186/s13058-015-0550-y)

Additional file 8: Kaplan-Meier curves for event-free survival analysis based on Rody's metagenes clustering. Rody's metagenes clustering was based on patients included in C2 and C3 (our cohort; see Figure 4A), and in C2' and C3' (GSE21653) (LIR, n = 70; HIR, n = 71).

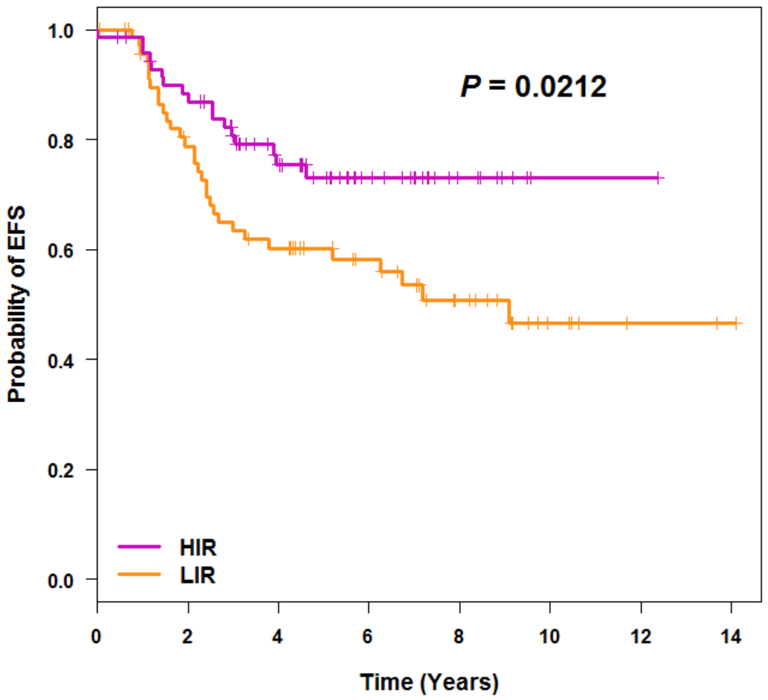

Supplement: Additional file 8: — Kaplan-Meier curves for event-free survival analysis based on Rody’s metagenes clustering. Rody’s metagenes clustering was based on patients included in C2 and C3 (our cohort; see Figure 4A), and in C2’ and C3’ (GSE21653) (low immune response (LIR), n = 70; high immune response (HIR), n = 71). [file 13058_2015_550_MOESM8_ESM.pdf]
