# Supplementary material for: Gene-expression molecular subtyping of triple-negative breast cancer tumours: importance of immune response
Source: Breast Cancer Res. 2015 Mar 20;17:43. doi: 10.1186/s13058-015-0550-y (PMC4389408; doi:10.1186/s13058-015-0550-y)
Supplement: Additional file 10: — Expression level of chosen genes between the three fuzzy clusters of our cohort. [file 13058_2015_550_MOESM10_ESM.pdf]

Additional file 10: Expression level of chosen genes between the three fuzzy clusters of our cohort.

Genes	Characteristics	*P*-value	*P*-value			Results[a]	Attended results[a]
			C2 vs C1	C3 vs C1	C3 vs C2		
AR	Luminal	< 0.0001	< 0.0001	< 0.0001	0.2139	1 > 2 = 3	1 > 2 = 3
ESR1		0.0033	0.0022	0.0715	0.4256	1 > 2 = 3[b]	
PGR		0.0018	0.0011	0.0626	0.3444	1 > 2 = 3[b]	
GATA3		< 0.0001	< 0.0001	0.0002	0.1150	1 > 2 = 3	
KRT18		< 0.0001	< 0.0001	< 0.0001	0.0544	1 > 2 = 3	
KRT19		0.0019	0.0316	0.0012	0.3143	1 > 2 = 3	
MUC1		< 0.0001	< 0.0001	< 0.0001	0.5087	1 > 2 = 3	
ERBB2	HER2-E	0.0133	0.3406	0.0109	0.1351	1 > 3	1 > 2 = 3
CDH3	Basal-like	0.0020	0.0015	0.1845	0.1401	1 < 2	1 < 2 = 3
EGFR		0.5695	-	-	-	-	
KIT		0.0003	0.0002	0.0144	0.4112	1 < 2 = 3	
KRT5		< 0.0001	< 0.0001	0.0032	0.1942	1 < 2 = 3	
KRT6A		0.3026	-	-	-	-	
KRT6B		< 0.0001	< 0.0001	0.0013	0.2904	1 < 2 = 3	
KRT14		0.0044	0.0029	0.1216	0.3256	1 < 2	
KRT17		< 0.0001	< 0.0001	0.0309	0.0087	1 < 3 < 2	
CDH1	Epithelial cell-cell	0.0084	0.3347	0.3943	0.0059	2 > 3	2 > 3
CGN	adhesion	0.1061	-	-	-	-	
CLDN3	Claudin-low	0.4679	-	-	-	-	
CLDN4		0.2535	-	-	-	-	
CLDN7		0.1226	-	-	-	-	
EPCAM		0.0009	0.1067	0.4122	0.0007	2 > 3	
OCLN		0.0023	0.1615	0.0016	0.0805	1 > 3 and 2 > 3[b]	
MKI67	Proliferation	< 0.0001	< 0.0001	< 0.0001	0.0061	1 < 3 < 2	1< 2 = 3 or
UBE2C		< 0.0001	< 0.0001	< 0.0001	0.0104	1 < 3 < 2	1 < 3 < 2
AURKA		< 0.0001	< 0.0001	< 0.0001	0.1180	1 < 2 = 3	
RACGAP1		< 0.0001	< 0.0001	0.0001	0.0234	1 < 3 < 2	
ABCA8	Breast stem cells	0.0093	0.0287	0.9590	0.0291	1 > 2 and 2 < 3	2 < 3
ALDH1A1		< 0.0001	< 0.0001	0.5478	< 0.0001	1 > 2 and 2 < 3	
CDH2	Epithelial-to-	0.0085	0.1300	0.7609	0.0085	2 > 3	2 < 3
FGF7	mesenchymal	0.0681	0.9998	0.1698	0.0788	2 < 3[b]	
FOXC2	transition (EMT)	0.9715	-	-	-	-	
SNAI1	Extracellular matrix	0.8216	-	-	-	-	
TGFB1		0.0006	0.2272	0.1818	0.0004	2 < 3	
TWIST1		0.0054	0.0037	0.0750	0.5211	1 > 2 and 1 > 3[b]	
VIM		0.5887	-	-	-	-	
ZEB1		0.0016	0.0011	0.1505	0.1488	1 > 2	
ITGA5	Cell migration	0.8629	-	-	-	-	2 < 3
MSN		< 0.0001	< 0.0001	< 0.0001	0.1933	1 < 2 = 3	
CD4	Immune system	< 0.0001	0.8179	0.0004	< 0.0001	1 < 3 and 2 < 3	2 < 3
CD79A	response	< 0.0001	0.0662	0.0010	< 0.0001	1 < 3 and 2 < 3	
CXCL2		0.0029	0.0425	0.9033	0.0042	1 > 2 and 2 < 3	
IL6		0.0021	0.9164	0.0082	0.0054	1 < 3 and 2 < 3	
STAT1		< 0.0001	0.5815	< 0.0001	< 0.0001	1 < 3 and 2 < 3	
VAV1		< 0.0001	0.4285	< 0.0001	< 0.0001	1 < 3 and 2 < 3	

[a]: expression level between clusters
[b]: trend: 0.05 < *P* (Tukey) < 0.10
