# Supplementary material for: Gene-expression molecular subtyping of triple-negative breast cancer tumours: importance of immune response
Source: Breast Cancer Res. 2015 Mar 20;17:43. doi: 10.1186/s13058-015-0550-y (PMC4389408; doi:10.1186/s13058-015-0550-y)
Supplement: Additional file 12: — Biological process gene ontology (GO) terms enrichment for our cohort. [file 13058_2015_550_MOESM12_ESM.pdf]

Additional file 12: Biological process GO terms enrichment for our cohort.

	H1	H2	H3	C1 vs C2	C1 vs C3	C2 vs C3	C1 vs C2-C3
EASE	epithelial cell differentiation	cell adhesion	immune response	M phase	immune response	immune response	immune response
	digestion	biological adhesion	lymphocyte activation	cell cycle	positive regulation of immune system process	defense response	defense response
	oxidation reduction	ectoderm development	defense response	organelle fission	defense response	positive regulation of immune system process	positive regulation of immune system process
	epithelium development	collagen catabolic process	cell activation	nuclear division	regulation of lymphocyte activation	cell activation	cell activation
	regulation of hormone levels	cell motion	leukocyte activation	mitosis	regulation of T cell activation	leukocyte activation	leukocyte activation
	hormone metabolic process	taxis	positive regulation of immune system process	cell cycle phase	regulation of cell activation	lymphocyte activation	lymphocyte activation
	urogenital system development	chemotaxis	cellular defense response	M phase of mitotic cell cycle	regulation of leukocyte activation	regulation of cell activation	regulation of cell activation
	xenobiotic metabolic process	skeletal system development	humoral immune response	cell cycle process	positive regulation of cell activation	regulation of lymphocyte activation	regulation of lymphocyte activation
	prostate gland development	neuron projection morphogenesis	inflammatory response	mitotic cell cycle	positive regulation of leukocyte activation	regulation of leukocyte activation	regulation of leukocyte activation
	response to xenobiotic stimulus	cell-cell adhesion	leukocyte mediated immunity	cell division	leukocyte activation	regulation of T cell activation	regulation of T cell activation
EASE synthesis	epithelial cell differentiation	cell adhesion	immune response	mitotic cell cycle	immune response	immune response	immune response
	digestion	ectoderm development					
	oxidation reduction	cell motion					
	hormone metabolic process						
ToppGene	epithelial cell differentiation	chemotaxis	immune response	M phase	immune response	immune response	immune response
	hormone metabolic process	taxis	regulation of immune system process	cell cycle phase	positive regulation of immune system process	defense response	defense response
	digestion	cell adhesion	regulation of immune response	M phase of mitotic cell cycle	regulation of immune system process	regulation of immune system process	regulation of immune system process
	lipid metabolic process	biological adhesion	positive regulation of immune system process	mitotic cell cycle	defense response	leukocyte activation	leukocyte activation
	epithelium development	locomotion	defense response	mitosis	regulation of immune response	cell activation	cell activation
	regulation of hormone levels	response to external stimulus	humoral immune response	nuclear division	leukocyte activation	regulation of immune response	regulation of immune response
	monocarboxylic acid metabolic process	cell-cell adhesion	lymphocyte activation	cell cycle process	lymphocyte activation	positive regulation of immune system process	positive regulation of immune system process
	cellular hormone metabolic process	extracellular structure organization	leukocyte activation	organelle fission	regulation of lymphocyte activation	lymphocyte activation	lymphocyte activation
	oxidation-reduction process	epithelium development	immune effector process	cell division	T cell activation	immune effector process	immune effector process
	xenobiotic metabolic process	central nervous system development	cell activation	cell cycle	regulation of leukocyte activation	positive regulation of immune response	positive regulation of immune response
ToppGene synthesis	epithelial cell differentiation	chemotaxis	immune response	mitotic cell cycle	immune response	immune response	immune response
	hormone metabolic process	cell adhesion					
	digestion	locomotion					
GOrilla	hormone metabolic process	anatomical structure development	immune response	cell cycle process	immune system process	immune system process	immune system process
	regulation of hormone levels	tissue development	immune system process	mitotic cell cycle	regulation of immune system process	regulation of immune system process	regulation of immune system process
	epithelial cell differentiation	locomotion	regulation of immune system process	cell division	positive regulation of immune system process	immune response	immune response
	cellular hormone metabolic process	chemotaxis	regulation of immune response	cell cycle	regulation of immune response	regulation of immune response	regulation of immune response
	xenobiotic metabolic process	taxis	humoral immune response	cell cycle phase	regulation of lymphocyte activation	defense response	defense response
	multicellular organismal process	developmental process	positive regulation of immune system process	mitotic prometaphase	defense response	positive regulation of immune system process	positive regulation of immune system process
	regulation of biological quality	collagen catabolic process	defense response	M phase of mitotic cell cycle	immune response	response to stimulus	response to stimulus
	monocarboxylic acid metabolic process	system development	response to stimulus	M phase	regulation of leukocyte activation	regulation of leukocyte activation	regulation of leukocyte activation
	single-multicellular organism process	granulocyte chemotaxis	complement activation	nuclear division	regulation of cell activation	regulation of cell activation	regulation of cell activation
	digestion	response to external stimulus	complement activation, classical pathway	mitosis	regulation of T cell activation	regulation of lymphocyte activation	regulation of lymphocyte activation
GOrilla synthesis	hormone metabolic process	anatomical structure development	immune response	mitotic cell cycle	immune response	immune response	immune response
	epithelial cell differentiation	locomotion					
		chemotaxis					
Global synthesis	epithelial cell differentiation	cell adhesion	immune response	mitotic cell cycle	immune response	immune response	immune response
	hormone metabolic process	locomotion					
		chemotaxis					
