# Supplementary material for: Gene-expression molecular subtyping of triple-negative breast cancer tumours: importance of immune response
Source: Breast Cancer Res. 2015 Mar 20;17:43. doi: 10.1186/s13058-015-0550-y (PMC4389408; doi:10.1186/s13058-015-0550-y)
Supplement: Additional file 13: — External validation process results. Eighty-seven triple negative (TN) patients of GSE21653 cohort were used for external validation. The same global process, as described for our cohort, was used and is described thereafter. [file 13058_2015_550_MOESM13_ESM.pdf]

Additional file 13: External validation process results. Eighty-seven TN patients of GSE21653 cohort were used for external validation. The same global process, as described for our cohort, was used and is described thereafter.

Clinicopathologic characteristics

We have selected 87 breast carcinomas that were shown to lack IHC expression of ER, PR and HER2. Contrary to our cohort, no significant age difference (P = 0.26) was found between the three clusters, named: C1', C2' and C3'. Histological grades were higher in C2 and C3 than in C1 (P = 0.0031). EFS did not show outcome difference according to cluster membership (P = 0.22) (Additional file 4). When GSE21653 was pooled with our 107 TN cohort, EFS results confirmed the trend found for our cohort (P = 0.01) (Additional file 4).

Gene-expression signatures

GES subtyping results are displayed in Figure 5 and Additional Table1.

Additional Table 1: GSE21653 gene-expression profiling in function of the three clusters.

**GES name**	**Subtypes**	**Cluster 1' (*n* = 27)**	**Cluster 2' (*n* = 28)**	**Cluster 3' (*n* = 32)**	***P*-value**
Sorlie's SSP	Basal-like	0	25	24	< 0.0001
	HER2-E	4	1	1	
	Luminal A	1	0	0	
	Luminal B	6	1	4	
	NBL	2	0	0	
	Unclassified	14	1	3	
Hu's SSP	Basal-like	0	25	27	< 0.0001
	HER2-E	3	0	1	
	Luminal A	12	0	0	
	Luminal B	1	0	0	
	NBL	2	2	1	
	Unclassified	9	1	3	
Parker's SSP = PAM50	Basal-like	0	27	30	< 0.0001
	HER2-E	7	0	0	
	Luminal A	7	0	0	
	Luminal B	6	0	1	
	NBL	4	1	1	
	Unclassified	3	0	0	
Proliferation score (mean[sd])		8.80 [1.13]	10.15 [0.69]	9.82 [0.68]	< 0.0001
TNBCtype	BL1	0	11	4	< 0.0001
	BL2	1	3	5	
	IM	1	0	18	
	LAR	17	0	0	
	M	1	7	0	
	MSL	5	2	3	
	Unclassified	2	5	2	
Teschendorff's GES	CC+	0	5	0	< 0.0001
	CC+/IR+	2	22	31	
	ECM+	6	0	1	
	IR+	1	0	0	
	SR+	13	0	0	
	Unclassified	5	1	0	
VEGF profile (mean[sd])		9.18 [0.42]	10.03 [0.56]	9.72 [0.60]	< 0.0001
Glycolysis profile (mean[sd])		11.81 [0.46]	12.18 [0.77]	12.08 [0.52]	0.0637
Claudin-low	Claudin-low	0	0	7	0.0009
	Other	27	28	25	

SSP, single sample predictor; GES, gene-expression signature

Single sample predictor annotation

These signatures showed that C1' essentially contained non basal-like subtypes. This cluster was mostly composed of luminal A and B subtypes, and unclassified tumours. In C1', PAM50 subtyping identified 48% of luminal subtypes (luminal A [26%] and B [22%]), no basal-like and 26% of HER2-E. This last subtype was not found in our cohort. C2' was an almost pure basal-like cluster whatever the SSP used. In C2', one patient was subtyped as normal breast-like by means of PAM50 and Hu's SSP and basal-like by Sorlie's SSP. GSE21653 bio-clinical data only indicated that histology of this tumour was different from infiltrating ductal carcinoma. C3' included mostly basal-like subtypes, but to a lesser extent than C2' (94% [PAM50 SSP], 84% [Hu's SSP] and 75% [Sorlie's SSP]); these results are comparable with those obtained for our cohort.

Proliferation score

Proliferation score was significantly lower in C1' compared to C2' (P < 0.0001) and C3' (P < 0.0001). No difference was found between C2' and C3' (P = 0.29).

TNBCtype

TNBCtype classification assigned a TNBC subtype to 90% of TN GSE21653 tumours. C1' was LAR-enriched (68% of classified patients) and LAR subtypes were exclusively assigned to this cluster. This result confirmed that C1' was not a basal-like cluster as shown with SSPs, and our results (100% of LAR in C1 and 61.5% of classified patients). C2' was BL1- and M-enriched (47.8% and 30%, respectively). BL1 and BL2 represented 61% of C2'. C3' was IM- and BL1/BL2-enriched (60% and 30%, respectively). MSL, characterized by low expression of proliferation genes compared to M, was mostly found in C1' (20%). Except one, all IM subtypes were included in C3'. Immune response distinguished C3' from C2'. These results are comparable with those found for our cohort.

Teschendorff's GES

Steroid hormone receptor (SR) subtypes were exclusively observed in C1'. CC+/IR+ were almost exclusively included in C2' (78%) and C3' (97%). This GES confirmed TNBCtype subtyping of C1': SR and LAR, respectively. Immune response was almost exclusively assigned to C2' and C3' and did not separate these two clusters. These results are comparable with those found for our cohort.

VEGF profile

This 13-GES showed that angiogenesis varied in function of the three clusters (P < 0.0001). Angiogenesis score was significantly lower in C1' compared to C2' (P < 0.0001) and C3' (P < 0.0006). A trend was found between C2' and C3', with C2' > C3'.

Glycolysis profile

Glycolysis score showed a trend according to clusters (P = 0.0637).

Claudin-low

Seven patients were subtyped as “claudin-low” (8%). The distribution was as follows: 0% in C1’ and C2’ and 22% (7/32) in C3’. So, C3’ was claudin-low-enriched but this subtype only represented around a fifth of its number. This result is comparable with the one found for our cohort (26% of C3).

Rody’s GES

For each immune module, metagene expressions were always significantly higher in C3’ compared to C2’ (data not shown). These results demonstrated that immune response could be considered as a hallmark of C3’. Ward’s hierarchical clustering method applied to these immune modules separated C2’ and C3’ patients in two clusters: one mostly composed of C2’ patients (95%) with low immune response (LIR) and the other composed of a majority (77.5%) of C3’ patients with high immune response (HIR) (Additional Figure 1). These results are comparable with those found for our cohort.

Considering C2’ and C3’ patients, EFS analyses were performed in function of Rody’s metagene subtypes. High expression of the majority of these metagenes was associated with a better outcome: MHC-II (P = 0.0272, HR = 0.61), IgG (P = 0.0024, HR = 0.70), Interferon (P = 0.0221, HR = 0.66), STAT1 (P = 0.0136, HR = 0.65), LCK (P = 0.0016, HR = 0.50). A trend was found for HCK (P = 0.08). MHC-I module was not associated with disease evolution (P = 0.26).

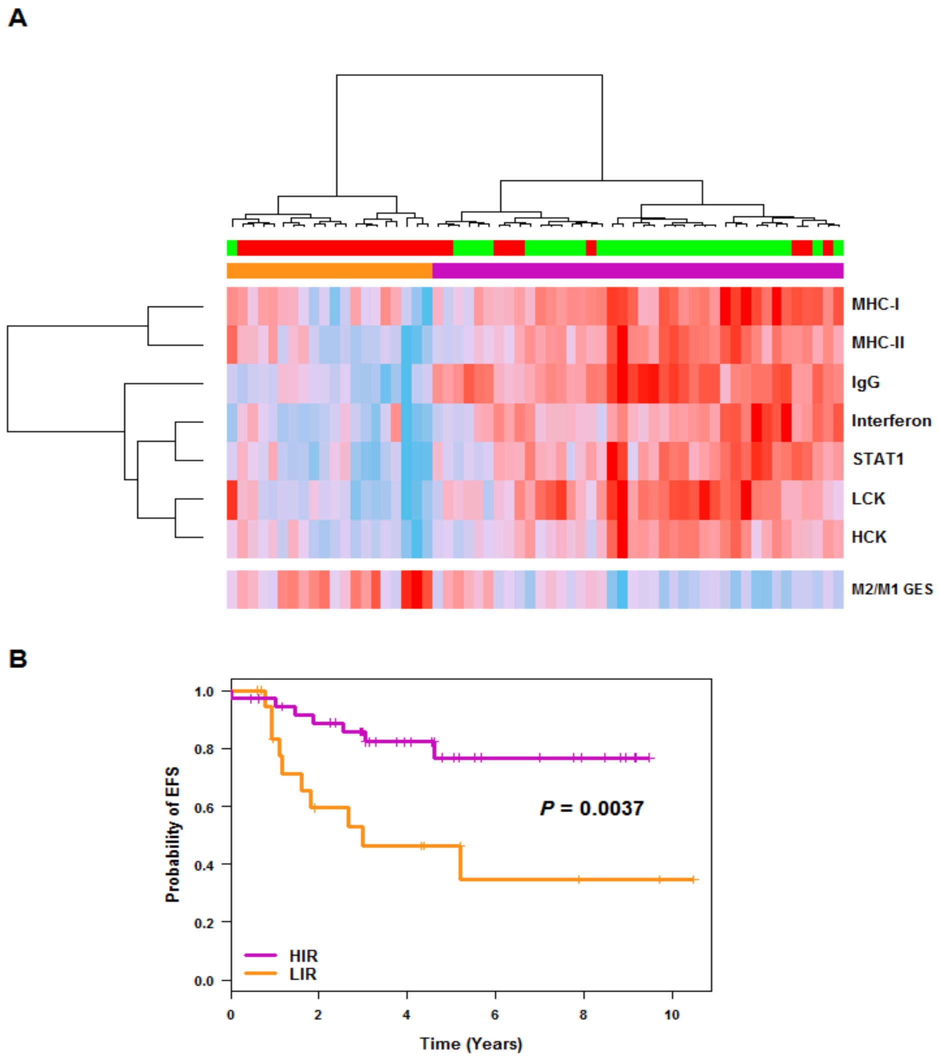

Additional Figure 1: C2'/C3' immune response dissection. (A) Ward's hierarchical clustering and heatmap showing the segregation of C2' (red) and C3' (green) patients in function of the seven Rody's metagenes (B lymphocytes [IgG]; macrophages and monocyte/myeloid lineage cells [HCK]; professional antigen-presenting cells [MHC-II]; T-cell [LCK]; cell types for presentation of intracellular antigens [MHC-I]; interferon signal transduction [STAT1]; interferon response [Interferon]) and M2/M1 GES: continuous colour scale, from minimum [deepskyblue] to maximum [red]. **(B)** Kaplan-Meier curves for event-free survival analysis of breast cancer patients with high immune response (HIR) and low immune response (LIR).

M2/M1 GES

Considering clusters C2' and C3', M2/M1 GES was significantly associated with bad outcome (EFS, $P = 0.03$), M2 protumorigenic macrophage genes were more expressed in C2' ($P < 0.0001$), which was characterized by LIR and bad outcome, and M1 tumour suppressor macrophage genes were more expressed in C3', which was characterized by HIR and better outcome. Of note, we did not find any correlation between VEGF score and M2 macrophage signature ($r = -0.10$; $P = 0.47$).

Single gene-expression intuitive approach

See Additional Table 2.

Additional Table 2: Expression level of chosen genes between the three fuzzy clusters of TN GSE21653 patients.

Genes	Characteristics	*P*-value	*P*-value			Results[a]	Attended results[a]	Validation[c]
			C2’ vs C1’	C3’ vs C1’	C3’ vs C2’			
AR	Luminal	< 0.0001	< 0.0001	< 0.0001	0.1120	1’ > 2’ = 3’	1’ > 2’ = 3’	Yes
ESR1		0.0883	-	-	-	-		No
PGR		0.0009	0.0015	0.0058	0.8438	1’ > 2’ = 3’		Yes
GATA3		< 0.0001	< 0.0001	< 0.0001	0.0550	1’ > 2’ = 3’		Yes
KRT18		< 0.0001	< 0.0001	< 0.0001	0.7206	1’ > 2’ = 3’		Yes
KRT19		0.0409	0.8345	0.1641	0.0425	2’ > 3’		No
MUC1		0.0001	0.0021	0.0002	0.8331	1’ > 2’ = 3’		Yes
ERBB2	HER2-E	0.0023	0.0032	0.0133	0.8256	1’ > 2’ = 3’	1’ > 2’ = 3’	No[d]
CDH3	Basal-like	0.0107	0.0141	0.0387	0.8814	1’ < 2’ = 3’	1’ < 2’ = 3’	No[e]
EGFR		0.2536	-	-	-	-		No
KIT		0.0001	0.0003	0.0006	0.9600	1’ < 2’ = 3’		Yes
KRT5		0.0002	0.0004	0.0020	0.7980	1’ < 2’ = 3’		Yes
KRT6A		0.0009	0.0010	0.0128	0.6014	1’ < 2’ = 3’		Yes
KRT6B		< 0.0001	< 0.0001	< 0.0001	0.3050	1’ < 2’ = 3’		Yes
KRT14		0.0009	0.0007	0.00283	0.3644	1’ < 2’ = 3’		No[f]
KRT17		< 0.0001	< 0.0001	0.0014	0.0810	1’ < 3’ < 2’[b]		Yes[b]
CDH1	Epithelial cell-cell adhesion	0.5595	-	-	-	-	2’ > 3’	No
CGN		0.5568	-	-	-	-		Yes
CLDN3		0.9116	-	-	-	-		No
CLDN4	Claudin-low	0.8214	-	-	-	-		No
CLDN7		0.1915	-	-	-	-		No
EPCAM		0.0366	0.3939	0.4294	0.0276	2’ > 3’		Yes
OCLN		0.0094	0.7008	0.0096	0.0774	1’ > 3’ and 2’ > 3’[b]		Yes
MKI67	Proliferation	< 0.0001	< 0.0001	0.0002	0.3319	1’ < 2’ = 3’	1’ < 2’ = 3’	Yes
UBE2C		< 0.0001	< 0.0001	0.0001	0.8777	1’ < 2’ = 3’	or	Yes
AURKA		0.0002	0.0025	0.0003	0.8481	1’ < 2’ = 3’	1’ < 3’ < 2’	Yes
RACGAP1		0.0004	0.0003	0.0163	0.3590	1’ < 2’ = 3’		Yes
ABCA8	Breast stem cells	0.8521	-	-	-	-	2’ < 3’	No
ALDH1A1		0.0006	0.0005	0.3290	0.0240	1’ > 2’ and 2’ < 3’		Yes
CDH2	Epithelial-to-mesenchymal transition (EMT)	0.8331	-	-	-	-	2’ < 3’	No
FGF7		0.3811	-	-	-	-		No
FOXC2		0.1171	-	-	-	-		No
SNAI1		0.7306	-	-	-	-		No
TGFB1	Extracellular matrix	0.0018	0.1166	0.2596	0.0011	2’ < 3’		Yes
TWIST1		0.8465	-	-	-	-		No
VIM		0.0073	0.0068	0.0565	0.6421	1’ < 2’		No
ZEB1		0.0009	0.0006	0.1877	0.0658	1’ > 2’ and 2’ < 3’[b]		No
ITGA5	Cell migration	0.7877	-	-	-	-	2’ < 3’	No
MSN		0.0004	0.0025	0.0010	0.9839	1’ < 2’ = 3’		No
CD4	Immune system response	0.0015	0.0206	0.7387	< 0.0015	1’ > 2’ and 2’ < 3’	2’ < 3’	Yes
CD79A		< 0.0001	0.0307	< 0.0001	< 0.0001	2’ < 1’ < 3’		Yes
CXCL2		0.0391	0.4771	0.0304	0.3471	1’ < 3’		No
IL6		< 0.0001	0.0041	< 0.0001	0.4132	1’ < 2’ and 1’ < 3’		No
STAT1		< 0.0001	0.9712	< 0.0001	< 0.0001	1’ < 3’ and 2’ < 3’		Yes
VAV1		< 0.0001	0.5396	0.0001	< 0.0001	1’ < 3’ and 2’ < 3’		Yes

[a]: expression level between clusters
[b]: trend: 0.05 < p (Tukey) < 0.10
[c]: comparable expression in our cohort and in GSE21653
[d]: SSP subtyping identified more HER2-E tumours in C1’ (GSE21653) contrary to C1 of our cohort (PAM50: 7 versus 1). This may explain *ERBB2* significant expression between C1’ and C2’-C3’. In our cohort, we only identified a and its constant level between the clusters of our cohort.
[e]: In GSE21653, P-cadherin RNA expression is in accordance with current knowledge.
[f]: 1 < 2 in both studies; 1 < 2 = 3 in GSE21653

GO biological process terms enrichment

GO biological process terms enrichment demonstrated that C1’ was characterized by digestion, steroid metabolic process, transport processes and oxidation-reduction process, C2’ by tissue development and cell differentiation, and C3’ by immune response (Additional Table 3). Comparing C2’ to C1’, C2’ was enriched in mitotic cell cycle genes. These results are in agreement with those found for our cohort.

Additional Table 3: Biological process GO terms enrichment for validation TN GSE21653 patients.

	H1	H2	H3	C1 vs C2	C1 vs C3	C2 vs C3	C1 vs C2-C3
EASE	digestion<br>secretion<br>cell-cell signaling<br>oxidation reduction<br>regulation of neurotransmitter levels<br>prostate gland development<br>steroid metabolic process<br>cell-cell adhesion<br>transmission of nerve impulse<br>neuron differentiation	ectoderm development<br>epidermis development<br>epithelial cell differentiation<br>epithelium development<br>peripheral nervous system development<br>keratinocyte differentiation<br>epidermal cell differentiation<br>cell motion<br>cell adhesion<br>biological adhesion	immune response<br>defense response<br>inflammatory response<br>chemotaxis<br>taxis<br>locomotory behavior<br>response to wounding<br>behavior<br>positive regulation of immune system process<br>cellular defense response	M phase<br>cell cycle phase<br>organelle fission<br>mitotic cell cycle<br>nuclear division<br>mitosis<br>cell cycle<br>M phase of mitotic cell cycle<br>cell cycle process<br>amine biosynthetic process	immune response<br>regulation of lymphocyte activation<br>regulation of leukocyte activation<br>regulation of T cell activation<br>regulation of cell activation<br>positive regulation of immune system process<br>leukocyte activation<br>cell activation<br>lymphocyte activation<br>positive regulation of leukocyte activation	immune response<br>lymphocyte activation<br>cell activation<br>leukocyte activation<br>T cell activation<br>positive regulation of immune system process<br>regulation of lymphocyte activation<br>regulation of cell activation<br>regulation of leukocyte activation<br>regulation of T cell activation	M phase<br>cell cycle phase<br>organelle fission<br>nuclear division<br>mitosis<br>M phase of mitotic cell cycle<br>mitotic cell cycle<br>cell cycle process<br>cell cycle<br>cell division
EASE synthesis	digestion<br>secretion<br>oxidation reduction<br>steroid metabolic process	tissue development<br>cell differentiation<br>cell motion<br>cell adhesion	immune response	mitotic cell cycle	immune response	immune response	cell cycle<br>mitotic cell cycle
ToppGene	digestion<br>cell fate commitment<br>lipid metabolic process<br>steroid metabolic process<br>hormone metabolic process<br>organic anion transport<br>oxidation-reduction process<br>anion transport<br>response to steroid hormone<br>response to lipid	epidermis development<br>skin development<br>epithelial cell differentiation<br>epithelium development<br>keratinocyte differentiation<br>epidermal cell differentiation<br>saliva secretion<br>keratinization<br>neuroblast proliferation<br>intermediate filament cytoskeleton organization	immune response<br>positive regulation of immune system process<br>regulation of immune system process<br>regulation of immune response<br>immune response-activating cell surface receptor signaling pathway<br>positive regulation of immune response<br>defense response<br>immune response-activating signal transduction<br>activation of immune response<br>lymphocyte activation	mitotic nuclear division<br>organelle fission<br>nuclear division<br>cell division<br>mitotic cell cycle<br>chromosome segregation<br>mitotic cell cycle process<br>cell cycle<br>O-glycan processing<br>protein O-linked glycosylation	immune response<br>regulation of immune system process<br>positive regulation of immune system process<br>leukocyte activation<br>lymphocyte activation<br>regulation of lymphocyte activation<br>T cell activation<br>regulation of immune response<br>positive regulation of immune response<br>regulation of T cell activation	immune response<br>leukocyte activation<br>lymphocyte activation<br>regulation of immune system process<br>cell activation<br>positive regulation of immune system process<br>regulation of immune response<br>T cell activation<br>defense response<br>regulation of lymphocyte activation	nuclear division<br>cell division<br>organelle fission<br>mitotic nuclear division<br>mitotic cell cycle process<br>mitotic cell cycle<br>cell cycle<br>chromosome segregation<br>cell cycle process<br>negative regulation of nuclear division
ToppGene synthesis	digestion<br>steroid metabolic process<br>anion transport<br>oxidation-reduction process	tissue development<br>cell differentiation	immune response	mitotic cell cycle	immune response	immune response	cell cycle<br>mitotic cell cycle
GOrilla	nitrogen compound transport<br>digestion<br>single-organism transport<br>secretion<br>organic anion transport<br>ion transport<br>appendage morphogenesis<br>limb morphogenesis<br>secretion by cell<br>oxidation-reduction process	epidermis development<br>epithelium development<br>tissue development<br>anatomical structure development<br>epithelial cell differentiation<br>developmental process<br>peripheral nervous system development<br>keratinization<br>epidermal cell differentiation<br>keratinocyte differentiation	immune system process<br>immune response<br>positive regulation of immune system process<br>regulation of immune system process<br>humoral immune response<br>defense response<br>regulation of immune response<br>positive regulation of immune response<br>immune response-activating cell surface receptor signaling pathway<br>immune response-activating signal transduction	organelle fission<br>nuclear division<br>mitotic nuclear division<br>single-organism process<br>mitotic cell cycle process<br>cell cycle process<br>O-glycan processing<br>protein O-linked glycosylation<br>microtubule polymerization or depolymerization<br>single-organism cellular process	immune system process<br>positive regulation of immune system process<br>regulation of immune system process<br>positive regulation of immune response<br>regulation of lymphocyte activation<br>regulation of immune response<br>immune response<br>regulation of T cell activation<br>regulation of leukocyte activation<br>immune response-activating signal transduction	immune system process<br>regulation of immune system process<br>positive regulation of immune system process<br>regulation of immune response<br>immune response<br>regulation of lymphocyte activation<br>defense response<br>regulation of leukocyte activation<br>regulation of cell activation<br>regulation of T cell activation	nuclear division<br>organelle fission<br>mitotic cell cycle process<br>mitotic nuclear division<br>cell cycle process<br>mitotic cell cycle<br>single-organism process<br>cell cycle<br>single-organism cellular process<br>negative regulation of nuclear division
GOrilla synthesis	transport and secretion processes<br>digestion<br>oxidation-reduction process	tissue development<br>cell differentiation	immune response	mitotic cell cycle	immune response	immune response	cell cycle<br>mitotic cell cycle
Global synthesis	digestion<br>steroid metabolic process<br>transport processes<br>oxidation-reduction process	tissue development<br>cell differentiation	immune response	mitotic cell cycle	immune response	immune response	cell cycle<br>mitotic cell cycle
